# Causal associations of male infertility with stroke: a two-sample Mendelian randomization study

**DOI:** 10.3389/fendo.2024.1338077

**Published:** 2024-04-15

**Authors:** Yutian Zhu, Xiyan Xin, Ziyang Yu, Siqi Guan, Jingshang Wang, Qiuning Liu, Lei Dong, Yang Ye

**Affiliations:** ^1^ Department of Traditional Chinese Medicine, Peking University Third Hospital, Beijing, China; ^2^ School of Traditional Chinese Medicine, Beijing University of Chinese Medicine, Beijing, China; ^3^ Department of Traditional Chinese Medicine, Beijing Obstetrics and Gynecology Hospital, Capital Medical University, Beijing, China; ^4^ Graduate School, Beijing University of Chinese Medicine, Beijing, China; ^5^ Key Laboratory of RNA Biology, Center for Big Data Research in Health, Institute of Biophysics, Chinese Academy of Sciences, Beijing, China

**Keywords:** male infertility, Mendelian randomization, stroke, small vessel stroke, GWAS

## Abstract

**Background:**

Stroke is a devastating global health issue, with high mortality and disability rates. The increasing prevalence of male infertility among reproductive-aged men has become a growing concern worldwide. However, the relationship between male infertility and stroke incidence remains uncertain. This study aimed to address this knowledge gap by employing a Mendelian randomization (MR) approach.

**Method:**

Utilizing genetic instrumental variables derived from a genome-wide association study (GWAS) on male infertility and stroke, a two-sample MR design was implemented. Five different analysis methods, with inverse-variance weighted as the primary approach, were used to examine the genetic causal associations between male infertility and various stroke subtypes. Heterogeneity analysis, pleiotropy tests, and leave-one-out validation were conducted to assess heterogeneity, evaluate pleiotropy, and ensure the robustness of the findings.

**Result:**

The results indicate a potential lower risk of small vessel stroke associated with male infertility (odds ratio, 95% confidence interval: 0.82, 0.68 to 0.99, *p*=0.044), although no significant impact on other stroke subtypes was observed. The study exhibited low heterogeneity and no apparent pleiotropy; however, the stability of the results was not optimal.

**Conclusion:**

Male infertility might potentially confer a protective effect against small vessel stroke risk. Caution is warranted due to potential confounding factors. Additional studies are necessary to confirm these findings and provide further validation.

## 1 Introduction

Stroke, also referred to as a cerebrovascular accident, is a significant and debilitating condition characterized by the sudden disruption of blood supply to the brain, resulting in impaired brain function. Annually, approximately 10.3 million new stroke cases are reported globally, leading to a loss of 113 million disability-adjusted life years (DALYs) (1). The lifetime risk of stroke after the age of 25 is estimated to be 24.9% globally, with men having a lifetime risk of 24.7% (2). Hypertension, smoking, diet, physical inactivity, and other factors contribute to the occurrence of stroke (3). Infertility, on the other hand, is the inability to conceive or achieve a successful pregnancy despite regular, unprotected sexual intercourse for a year or more (4). It affects a vast population, with an estimated 186 million individuals worldwide experiencing infertility, impacting 8-12% of couples in their reproductive age (5). The causes of infertility can be attributed to various factors, including physiological, hormonal, genetic, and lifestyle factors. Male infertility, specifically, is a prevalent condition that hinders a man’s ability to contribute to conception or father a child due to various reproductive factors (6). It constitutes a significant reproductive health concern, affecting numerous couples globally and impeding their ability to achieve pregnancy and build a family (7).

Previous studies have shown a certain association between fertility issues and an increased risk of stroke in women. For instance, a large-scale cohort study found that female infertility was linked to a higher risk of non-fatal stroke (8). Another prospective cohort study revealed that women with endometriosis have an elevated risk of stroke (9). Nevertheless, contrasting these findings, there are studies that suggest an uncertain relationship between female infertility and stroke (10). It is worth noting that infertility and stroke are prevalent conditions that affect men as well, and it remains uncertain whether there is a causal relationship between the two.

Mendelian randomization (MR) is a robust technique that employs genetic variants as instrumental variables to explore causal relationships between exposures and outcomes, effectively addressing biases and confounding factors (11). By capitalizing on the random assortment of genetic variants during meiosis, MR offers valuable insights into disease pathogenesis. In our study, we employed a two-sample MR approach to investigate the causal association between male infertility and stroke. This research aims to uncover novel genetic perspectives on the link between male infertility and stroke, ultimately enhancing our understanding of the underlying mechanisms involved in stroke pathogenesis.

## 2 Materials and methods

### 2.1 Study overview

**Figure 1:**
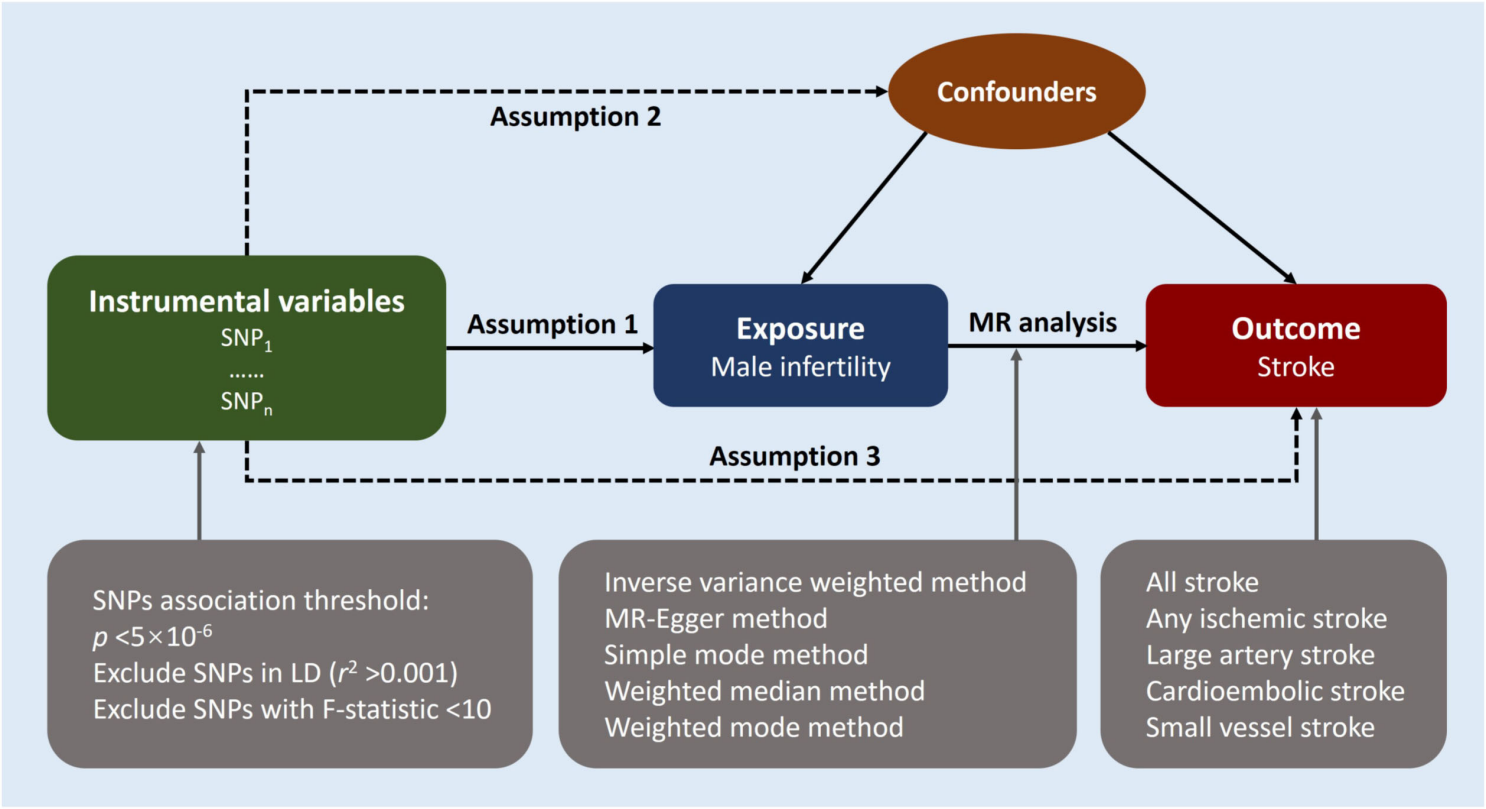
Study design overview. Mendelian randomization design relies on three key assumptions. The first assumption requires the genetic variants utilized as instrumental variables to be reliably associated with the exposure of interest. The second assumption states that these genetic variants should not be associated with any potential confounders, thereby ensuring that the instrumental variables are specific to the exposure. Lastly, the third assumption presupposes that the selected genetic variants influence the risk of the outcome solely through the risk factor being investigated, excluding potential alternative causal pathways. These assumptions are fundamental in establishing a causal relationship between male infertility and the risk of stroke using Mendelian randomization. LD indicates linkage disequilibrium; MR, Mendelian randomization; SNP, single-nucleotide polymorphisms.

We performed a two-sample MR approach to ascertain the potential causal relationship between male infertility and stroke. An overview of this study design is shown in **Figure 1**. The key assumptions of MR are as follows: (1) strong predictive power of the instruments for the exposures, (2) absence of confounding between the instruments and the exposure-outcome association as causes or consequences, and (3) independence of the instruments from the outcome when considering the exposure of interest (12). We implemented stringent selection criteria to ensure that the identified genetic variables met the necessary requirements for our MR study. Analysis encompassed the use of five distinct MR methods, with the inverse-variance weighted (IVW) method serving as the primary approach. In addition, we conducted subtype-specific analyses for stroke. Furthermore, the MR results were evaluated through various analysis methods including heterogeneity analysis, pleiotropy tests, and leave-one-out sensitivity analysis. Since our study solely utilized publicly available summary data, no ethical approval was required for this research.

### 2.2 Genetic instrumental variables

The genetic variables were sourced from the large publicly available genome-wide association studies (GWAS) conducted on diverse populations. A summary of the GWAS utilized in our study is shown in **Table 1**. Based on the FinnGen R9 dataset, we selected single nucleotide polymorphisms (SNPs) associated with male infertility to create our genetic instruments. The FinnGen R9 comprises genomic data collected from more than 300,000 Finnish individuals, making it one of the largest genetic databases of its kind (13). From the FinnGen R9 cohort, we included 13,142 participants with male infertility as cases, while 107,564 participants were chosen as controls. We screened the SNPs to identify those that exhibited a strong association with male infertility. This screening involved considering a genome-wide significance level of *p <*5×10^−6^, ensuring that only highly significant SNPs were included in the analysis (14). To ensure the independence of SNPs for MR analyses, a linkage disequilibrium clumping algorithm was employed to identify SNPs that exhibit minimal correlation with each other (*r* ^2^<0.001) (15). Furthermore, the *F* statistic was used to evaluate the potential bias caused by weak instruments. An *F* statistic value exceeding 10 signifies the presence of strong genetic instrumental variables (16). An analysis was also conducted to assess the correlation between genetic instruments and various types of strokes.

**Table 1:** GWASs used in this study.

Traits	GWASs	Sample size(cases/controls)
Exposure		
Male infertility	FinnGen R9	13,142/107,564
Outcomes		
AS	MEGASTROKE	67,162/454,450
AIS	MEGASTROKE	60,341
LAS	MEGASTROKE	6,688
CES	MEGASTROKE	9,006
SVS	MEGASTROKE	11,710

AIS, any ischemic stroke; AS, all stroke; CES, cardioembolic stroke; GWAS, genome-wide association studies; LAS, large artery stroke; SVS, small vessel stroke.

### 2.3 Data sources for stroke

The genetic data for stroke were extracted from the MEGASTROKE project, initiated by the International Stroke Genetics Consortium, with a comprehensive description of the project available in previous publications (17). Besides the overall stroke (all stroke, AS), the MEGASTROKE Consortium further categorizes stroke into four subtypes, namely any ischemic stroke (AIS), large artery stroke (LAS), cardioembolic stroke (CES), and small vessel stroke (SVS) (18). This study consisting of 67,162 stroke cases and 454,450 controls. Among the AS cases, 60,341 were subclassified as AIS, 6,688 as LAS, 9,006 as CES, and 11,710 as SVS (17, 19).

### 2.4 Statistical analysis

The primary analysis in the MR study involved IVW regression analysis, assuming the absence of invalid genetic instruments (20). This study also utilized four other MR analysis methods, namely MR-Egger, weighted median, simple mode, and weighted mode. In MR, the IVW method is commonly used, combining causal estimates from multiple genetic variants by weighting them based on their inverse variances, assuming valid instruments and no directional pleiotropy (21). MR-Egger regression estimates both causal effects and pleiotropy bias simultaneously, accommodating variants violating the assumption of no pleiotropy (22). The weighted median method takes the median of individual causal estimates, providing consistent estimates with more than 50% weight from valid instruments, even with invalid instruments or moderate heterogeneity (23). The simple mode method calculates the most frequent causal estimate among genetic variants, valid with no heterogeneity or biased causal estimates (24). The weighted mode method combines frequent causal estimates with proportional weights for addressing heterogeneity (25).

The results are reported in terms of odds ratios (ORs) and β values, which provide insights into the risk of stroke. An OR value greater than 1 suggests that the exposure factors are adverse factors for the outcome, while an OR value less than 1 indicates favorable factors (26). Similarly, β values less than 0 indicate that the exposure factors are risk factors for the outcome, while β values greater than 0 signify favorable factors (27). The MR analyses were conducted using the “TwoSampleMR” package in R (version 4.21, R Development Core Team, Vienna, Austria) (28). Heterogeneity was evaluated using the IVW and MR-Egger tests, with a *p*-value less than 0.05 indicating the presence of heterogeneity in the study (29). We also employed Cochrane’s *Q* statistic to assess the heterogeneity among the SNP-specific causal estimates (30). The MR-Egger intercept test was performed to assess the presence of pleiotropy in the data and evaluate the robustness of the results. A *p*-value less than 0.05 indicates the presence of pleiotropy in the data (31). Furthermore, for sensitivity analysis, we employed the leave-one-out method, systematically removing each SNP individually and repeating the IVW analysis. This approach enabled us to evaluate the potential impact of each SNP on the observed causal effects (32).

## 3 Results

### 3.1 Genetic instruments for male infertility

After a thorough screening process, a total of 24 SNPs associated with male infertility were identified from the FinnGen project and included in this study. Notably, all of these SNPs exhibited *F* statistics greater than 20, suggesting the exclusion of weak instrumental variable bias. **Supplementary Tables S1, S2** provide detailed information regarding the instrumental variables used in the study.

### 3.2 MR analyses

**Figure 2:**
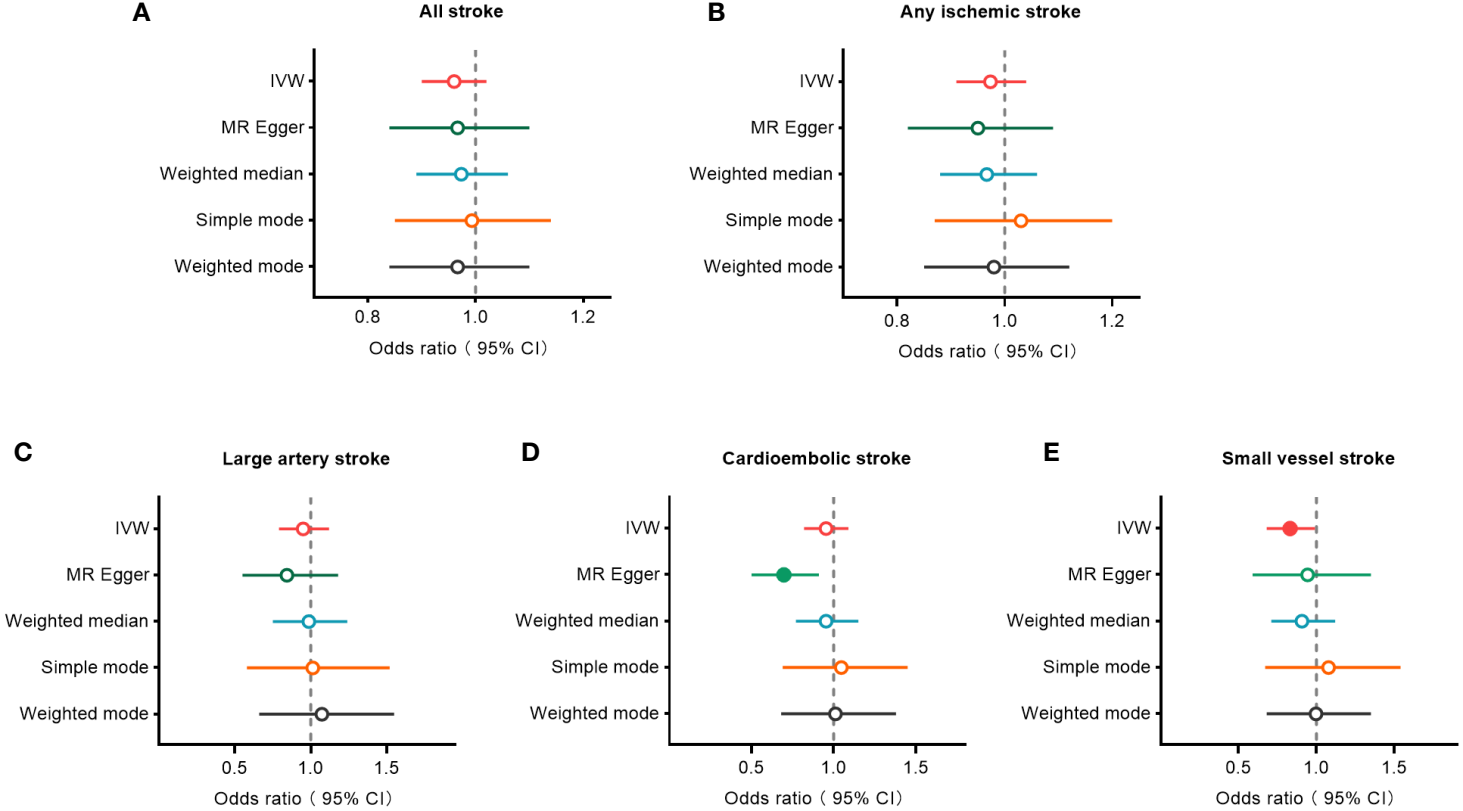
Forest plots to visualize causal effect of male infertility on stroke and its subtypes. All stroke **(A)**, any ischemic stroke **(B)**, large artery stroke **(C)**, cardioembolic stroke **(D)**, and small vessel stroke **(E)**. An open circle indicates no statistically significant difference in the results, while a solid circle indicates a significant difference. CI indicates confidence interval; IVW, inverse-variance weighted; MR, Mendelian randomization.

**Figure 3:**
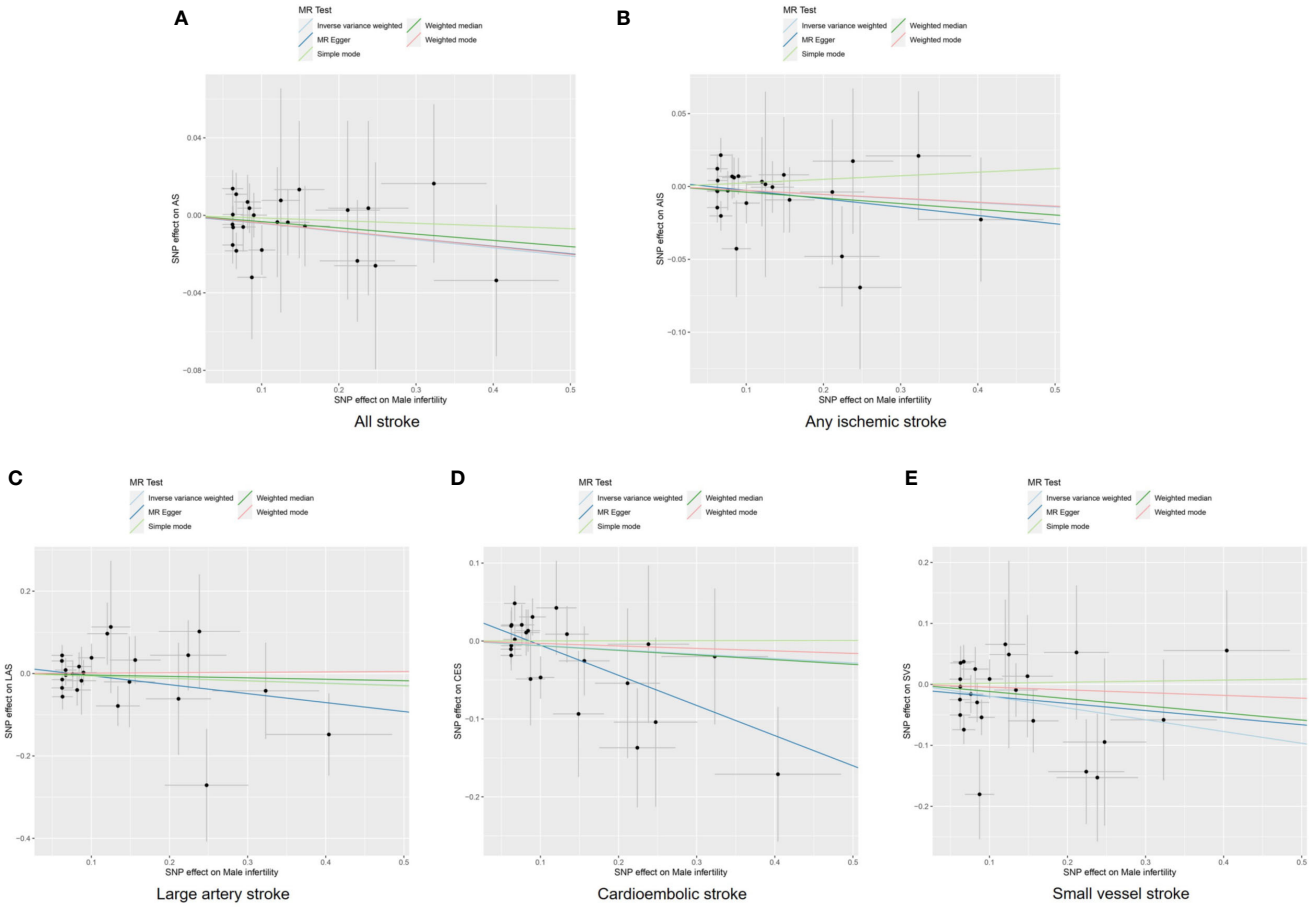
Scatter plots to visualize causal effect of male infertility on stroke and its subtypes. All stroke **(A)**, any ischemic stroke **(B)**, large artery stroke **(C)**, cardioembolic stroke **(D)**, and small vessel stroke **(E)**. AIS indicates any ischemic stroke; AS, all stroke; CES, cardioembolic stroke; LAS, large artery stroke; MR, Mendelian randomization; SNP, single nucleotide polymorphisms; SVS, small vessel stroke.

The IVW, MR-Egger, weighted median, simple mode, and weighted mode approaches were employed to estimate the causal associations between genetically predicted male infertility and the risk of stroke. We found no causal relationship between male infertility and the overall incidence of stroke or ischemic stroke using all the methods (**Table 2**, **Figures 2**, **3**). In the analysis of ischemic stroke subtypes, we observed no association between male infertility and large vessel stroke (OR=0.94, 95% CI=0.79-1.12, *p*=0.518) or cardioembolic stroke (OR=0.95, 95% CI=0.82-1.09, *p*=0.424) when using the IVW method. However, there was a causal relationship indicating a decreased risk of small vessel stroke associated with male infertility (OR=0.82, 95% CI=0.68-0.99, *p*=0.044, IVW method). Using MR-Egger, we found evidence of causal relationships between decreased risk of cardioembolic stroke and male infertility (OR=0.68, 95% CI=0.50-0.91, *p*=0.018). Forest plots illustrating the effect estimates for the causal relationship between male infertility and the risk of various stroke types at each SNP level were presented in **Supplementary Figures S1-S5**.

**Table 2:** Mendelian randomization results of causal effects of male infertility on risk of stroke and its subtypes.

Method	SNPs, n	OR	95% CI	*p* value
All stroke				
IVW	24	0.96	0.90-1.02	0.188
MR Egger	24	0.96	0.84-1.10	0.579
Weighted median	24	0.97	0.89-1.06	0.472
Simple mode	24	0.99	0.85-1.14	0.855
Weighted mode	24	0.96	0.84-1.10	0.567
Any ischemic stroke				
IVW	24	0.97	0.91-1.04	0.414
MR Egger	24	0.94	0.82-1.09	0.455
Weighted median	24	0.96	0.88-1.06	0.415
Simple mode	24	1.02	0.87-1.20	0.768
Weighted mode	24	0.97	0.85-1.12	0.708
Large artery stroke				
IVW	24	0.94	0.79-1.12	0.518
MR Egger	24	0.80	0.55-1.18	0.278
Weighted median	24	0.97	0.75-1.24	0.784
Simple mode	24	0.94	0.58-1.52	0.810
Weighted mode	24	1.01	0.66-1.55	0.964
Cardioembolic stroke				
IVW	23	0.95	0.82-1.09	0.424
MR Egger	23	0.68	0.50-0.91	0.018
Weighted median	23	0.94	0.77-1.15	0.564
Simple mode	23	1.00	0.69-1.45	0.996
Weighted mode	23	0.97	0.68-1.38	0.862
Small vessel stroke				
IVW	24	0.82	0.68-0.99	0.044
MR Egger	24	0.89	0.59-1.35	0.589
Weighted median	24	0.89	0.71-1.12	0.323
Simple mode	24	1.02	0.67-1.54	0.935
Weighted mode	24	0.96	0.68-1.35	0.798

CI, confidence interval; IVW, inverse-variance-weighted; MR, Mendelian randomization; OR, odds ratio; SNP, single nucleotide polymorphism.

### 3.3 Sensitivity analyses

**Figure 4:**
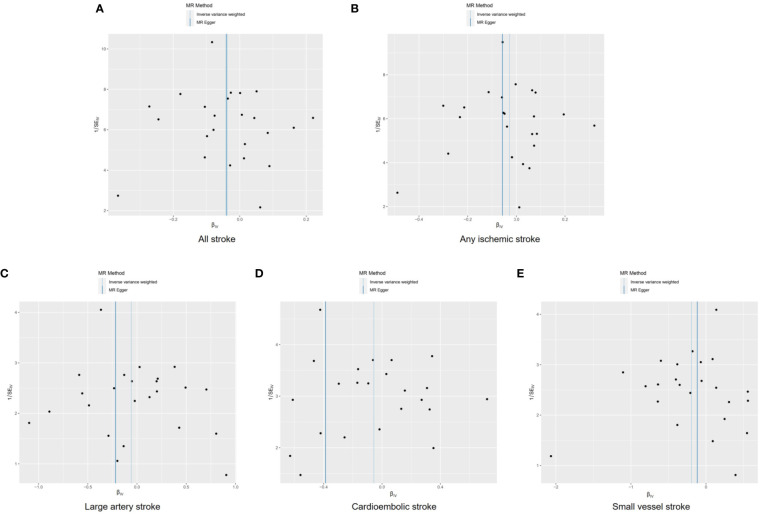
Funnel plots to visualize overall heterogeneity of Mendelian randomization estimates for the effect of male infertility on stroke and its subtypes. All stroke **(A)**, any ischemic stroke **(B)**, large artery stroke **(C)**, cardioembolic stroke **(D)**, and small vessel stroke **(E)**. MR indicates Mendelian randomization; SE, standard error.

Funnel plots were used to visualize the individual Wald ratios of each SNP plotted against their precision, enabling the detection of potential directional horizontal pleiotropy through the observation of asymmetry. No significant asymmetry was observed in the IVW method’s funnel plots for various stroke types (**Figure 4**). However, when using the MR-Egger approach, noticeable asymmetry was observed in the funnel plot for cardioembolic stroke. It should be noted that assessing the symmetry of funnel plots can be challenging when the number of genetic instruments is limited. There were no statistically significant differences in heterogeneity among the subtypes of stroke (*Q*>0.05; **Supplementary Table S3**), indicating low heterogeneity in this study.

The intercept of the MR-Egger analysis did not provide substantial evidence of directional pleiotropy in overall stroke, ischemic stroke, large vessel stroke, or small vessel stroke (*p*=0.964, *p*=0.667, *p*=0.368, and *p*=0.688, respectively; **Supplementary Table S4**). However, significant evidence of directional pleiotropy was observed in cardioembolic stroke (*p*=0.023).

**Figure 5:**
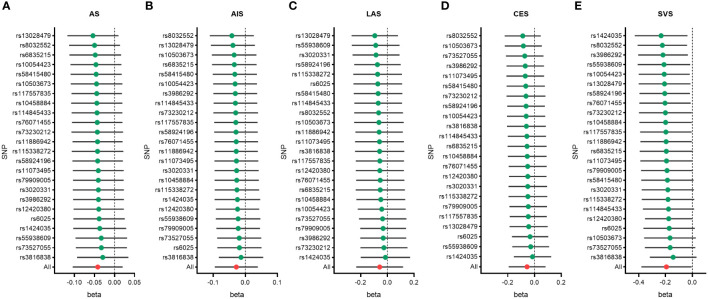
Leave-one-out analysis of genetic risk of male infertility on stroke and its subtypes. All stroke **(A)**, any ischemic stroke **(B)**, large artery stroke **(C)**, cardioembolic stroke **(D)**, and small vessel stroke **(E)**. AIS indicates any ischemic stroke; AS, all stroke; CES, cardioembolic stroke; LAS, large artery stroke; SNP, single nucleotide polymorphisms; SVS, small vessel stroke.

To identify potential influencing SNPs that could bias the causal association, we conducted leave-one-out analyses using IVW method. We found that removing any individual SNP did not significantly affect the final results in terms of overall stroke, ischemic stroke, large vessel stroke, and cardioembolic stroke (**Figure 5**, **Supplementary Tables S5-S9**). However, in the case of small vessel stroke, removing several SNPs such as rs3816838, rs73527055, rs10503673, among others, could potentially result in no statistically significant difference in the final outcome.

## 4 Discussion

This groundbreaking MR study aimed to examine the potential relationship between genetic variations in male infertility and the risk of stroke. Utilizing comprehensive genetic association data on stroke risk, our investigation revealed intriguing findings. We discovered that male infertility might potentially confer a protective effect against small vessel stroke risk. However, it is important to note that no similar associations were observed with other subtypes of stroke.

Our study provides novel evidence suggesting that male infertility may offer a protective effect against the risk of stroke, challenging existing perceptions. Importantly, to our knowledge, there have been no prior studies specifically examining the relationship between male infertility and stroke risk. However, studies on female infertility and stroke risk shed some light on this topic. For instance, a comprehensive analysis combining data from eight prospective cohort studies indicated an increased risk of non-fatal stroke associated with female infertility (8). Similarly, another study demonstrated that a history of infertility independently increased the risk of perinatal arterial ischemic stroke (33). Nonetheless, there are also studies indicating no significant association between female infertility and stroke risk. For example, research has shown that while women with endometriosis face an increased risk of stroke, a history of infertility itself is not associated with an elevated stroke risk (9). Likewise, a meta-analysis incorporating 18 clinical studies revealed a heightened risk of stroke among women with a history of miscarriage or stillbirth, yet failed to establish a notable association between infertility and stroke risk (10). Additionally, there are studies aligning with our findings. A study exploring the relationship between infertility medications and ischemic stroke found that women who utilized infertility medications had a lower risk of developing this condition (34).

Due to variations in the etiology and pathophysiology of different stroke subtypes, the impact of male infertility on these subtypes may differ. This study has revealed a previously unmentioned association between male infertility and a reduced risk of small vessel stroke. This effect could potentially be attributed to commonly used medications for male infertility, such as L-carnitine and vitamin C, which are known to ameliorate microcirculatory dysfunction and possibly prevent the occurrence of small vessel stroke. In an animal experiment, researchers found that propionyl-L-carnitine could prevent leakage and leukocyte adhesion, while maintaining capillary perfusion, thereby mitigating vascular ischemia-reperfusion injury (35). Furthermore, clinical studies have indicated that propionyl-L-carnitine increases post-ischemic blood flow, leading to improved microcirculatory dysfunction (36). Vitamin C has also demonstrated the ability to restore coronary microvascular responsiveness in smokers (37), and its supplementation has been found beneficial for peripheral tissue perfusion and microvascular reactivity in patients with septic shock (38).

The potential reasons behind the observed association between male infertility and a reduced risk of stroke are likely to be influenced by multiple factors. Research has demonstrated that women who utilize infertility medications have a lower risk of ischemic stroke, thus implying that treatments for female infertility may also mitigate the risk of stroke (34). Similarly, it is plausible that medications used to treat male infertility could have a similar effect on reducing the risk of stroke. For example, commonly used hormones for the treatment of male infertility, such as follicle-stimulating hormone (FSH) (39), human chorionic gonadotropin (hCG) (40), and gonadotropin-releasing hormone (GnRH) (41), are utilized to stimulate the release of testosterone and the production of sperm. Considering the existing evidence suggesting a potential negative correlation between testosterone levels and the risk of stroke (42, 43), it is plausible that hormones used for male infertility might have a potential effect in lowering the risk of stroke. Furthermore, adopting healthy lifestyle practices is an integral component of male infertility treatment. Factors such as weight loss, regular physical exercise, and refraining from smoking and excessive alcohol consumption, which are known to be beneficial for addressing male infertility, may also contribute to reducing the risk of stroke (44–46).

This study possesses several notable strengths. Firstly, we utilized a combination of five different methods for MR analysis, each method having distinct advantages and limitations. By incorporating the findings from these diverse approaches, we were able to conduct a comprehensive and well-rounded analysis. Secondly, we adopted the MEGASTROKE classification to categorize strokes into five types, facilitating a comprehensive understanding of how male infertility impacts different stroke subtypes. This classification system enhanced the precision of our findings and their applicability to clinical practice. Thirdly, our study exhibited low heterogeneity, suggesting a consistency in the results obtained. Furthermore, we did not observe any inherent multiple effects that could potentially confound our conclusions. Lastly, the novel focus of our research provides robust scientific guidance for effectively managing and preventing the future risk of stroke among individuals with male infertility. By shedding light on this underexplored aspect, our study offers valuable insights for healthcare professionals and contributes to the development of targeted preventive strategies.

This study acknowledges several limitations that should be considered. Firstly, male infertility has various subtypes, such as spermatogenic dysfunction, obstructive azoospermia, idiopathic male infertility, among others. However, we did not conduct separate analyses for these different subtypes, limiting our ability to determine the causality between specific types of male infertility and stroke risk. Secondly, we adopted a more relaxed threshold (*p*<5×10^-6^) for selecting genetic instrumental variables from GWAS, instead of the commonly used threshold (*p*<5×10^-8^) in the literature (47). While this allowed us to include more instrumental variables for a robust MR analysis, it may increase the risk of false-positive results. Thirdly, our sensitivity analyses highlighted that certain individual SNPs might potentially impact the results of the MR analysis, indicating a degree of instability in our findings. Fourthly, we did not analyze the treatment profiles of stroke patients, which could have provided valuable insights into whether fertility medications influence stroke risk.

Finally, as this study is based on GWAS analysis, it is unclear whether our conclusions can be generalized to different populations or ethnicities.

## 5 Conclusion

To our knowledge, this is the first study to establish a potential association between male infertility and the risk of stroke. Our findings indicate a lower risk of small vessel stroke among individuals with male infertility. However, it is crucial to exercise caution when interpreting these results, considering the limitations of the MR method, variations among different subtypes of male infertility, and the possibility of confounding factors. Additional research is necessary to validate the relationship between male infertility and stroke in the future.

## Data availability statement

The original contributions presented in the study are included in the article/**Supplementary Material**. Further inquiries can be directed to the corresponding authors.

## Author contributions

YZ: Formal analysis, Funding acquisition, Investigation, Methodology, Writing – original draft. XX: Formal analysis, Methodology, Writing – review & editing. ZY: Formal analysis, Investigation, Methodology, Writing – original draft. SG: Investigation, Methodology, Writing – review & editing. JW: Investigation, Software, Writing – review & editing. QL: Methodology, Software, Writing – review & editing. LD: Conceptualization, Formal analysis, Supervision, Writing – review & editing. YY: Conceptualization, Supervision, Writing – original draft, Writing – review & editing.
