# Supplementary material for: Causal associations of male infertility with stroke: a two-sample Mendelian randomization study
Source: Front Endocrinol (Lausanne). 2024 Apr 15;15:1338077. doi: 10.3389/fendo.2024.1338077 (PMC11056502; doi:10.3389/fendo.2024.1338077)
Supplement: Supplementary file 1 [file DataSheet_1.pdf]

Supplementary Material

Supplementary Table 1. Instrumental variables used in the study.

Supplementary Table 2. Association between SNPs and different types of strokes.

Supplementary Table 3. Results of the heterogeneity tests conducted in the MR analysis.

Supplementary Table 4. Results of the pleiotropy tests conducted in the MR analysis.

Supplementary Table 5. Results of the leave-one-out sensitivity analyses for all stroke.

Supplementary Table 6. Results of the leave-one-out sensitivity analyses for any ischemic stroke.

Supplementary Table 7. Results of the leave-one-out sensitivity analyses for large artery stroke.

Supplementary Table 8. Results of the leave-one-out sensitivity analyses for cardioembolic stroke.

Supplementary Table 9. Results of the leave-one-out sensitivity analyses for small vessel stroke.

Supplementary Table 10. The full list of MEGASTROKE authors.

Supplementary Figure 1. Forest plot of effect estimates for the causal relationship between male infertility and all stroke risk at each SNP level.

Supplementary Figure 2. Forest plot of effect estimates for the causal relationship between male infertility and any ischemic stroke risk at each SNP level.

Supplementary Figure 3. Forest plot of effect estimates for the causal relationship between male infertility and large artery stroke risk at each SNP level.

Supplementary Figure 4. Forest plot of effect estimates for the causal relationship between male infertility and cardioembolic stroke risk at each SNP level.

Supplementary Figure 5. Forest plot of effect estimates for the causal relationship between male infertility and small vessel stroke risk at each SNP level.

Table S1. Instrumental variables used in the study.

SNP	MAF	R^2	*F* statistic	*p* value
rs6025	0.020875	0.000175	21.08117	4.40E-06
rs13028479	0.405359	0.000177	21.4012	3.72E-06
rs114845433	0.014401	0.000174	20.95868	4.69E-06
rs11886942	0.073174	0.000208	25.15322	5.29E-07
rs73230212	0.063737	0.000190	22.98676	1.63E-06
rs3986292	0.45442	0.000188	22.74229	1.85E-06
rs6835215	0.008501	0.000186	22.40089	2.21E-06
rs10054423	0.169033	0.000181	21.89196	2.88E-06
rs115338272	0.039863	0.000191	23.0735	1.56E-06
rs3020331	0.482914	0.000185	22.34999	2.27E-06
rs58415480	0.230483	0.000242	29.25152	6.36E-08
rs10503673	0.195325	0.000230	27.81466	1.33E-07
rs55938609	0.164037	0.000272	32.82064	1.01E-08
rs10458884	0.462399	0.000186	22.46578	2.14E-06
rs12420380	0.142268	0.000180	21.7612	3.09E-06
rs73527055	0.442542	0.000183	22.05934	2.64E-06
rs3816838	0.268847	0.000173	20.88811	4.87E-06
rs76071455	0.028717	0.000214	25.79161	3.80E-07
rs79909005	0.018235	0.000178	21.44435	3.64E-06
rs8032552	0.357629	0.000198	23.88909	1.02E-06
rs11073495	0.224567	0.000186	22.46844	2.14E-06
rs1424035	0.005578	0.000207	24.96911	5.82E-07
rs117557835	0.045874	0.000173	20.84978	4.97E-06
rs58924196	0.066399	0.000181	21.79337	3.04E-06

EAF, effect allele frequency; MAF, minor allele frequency; SNP, single nucleotide polymorphism.

Table S2. Association between SNPs and different types of strokes.

SNP	*p* value-AS	*p* value-AIS	*p* value-LAS	*p* value-CES	*p* value-SVS
rs10054423	0.6209	0.652	0.2914	0.7149	0.487
rs10458884	0.9634	0.7456	0.5612	0.7596	0.8735
rs10503673	0.9953	0.5702	0.9374	0.1945	0.0636
rs11073495	0.6366	0.8293	0.9598	0.4256	0.6091
rs114845433	0.9337	0.7273	0.462	0.9677	0.1455
rs115338272	0.7847	0.6798	0.5726	0.5647	0.2495
rs117557835	0.7077	0.8403	0.8547	0.2465	0.8933
rs11886942	0.8938	0.9811	0.4806	-	0.7497
rs12420380	0.3162	0.1985	0.8327	0.4108	0.0142
rs13028479	0.1454	0.2241	0.0813	0.3393	0.7308
rs1424035	0.3908	0.5935	0.1378	0.0478	0.5741
rs3020331	0.6110	0.7648	0.2164	0.5813	0.2759
rs3816838	0.0511	0.0481	0.8902	0.9172	0.0016
rs3986292	0.5746	0.7335	0.0703	0.3714	0.2018
rs55938609	0.1656	0.4122	0.2639	0.0852	0.7839
rs58415480	0.7701	0.6576	0.6232	0.6269	0.3608
rs58924196	0.9028	0.9146	0.198	0.4811	0.3695
rs6025	0.4533	0.1624	0.6003	0.0733	0.0955
rs6835215	0.6882	0.6347	0.7202	0.8198	0.5563
rs73230212	0.8355	0.9804	0.1046	0.8087	0.8331
rs73527055	0.1113	0.1595	0.1826	0.3316	0.0389
rs76071455	0.9539	0.9395	0.6519	0.573	0.6339
rs79909005	0.6263	0.2172	0.0475	0.3373	0.4905
rs8032552	0.3198	0.07007	0.7695	0.0343	0.1713

AIS, any ischemic stroke; AS, all stroke; CES, cardioembolic stroke; LAS, large artery stroke; SNP, single nucleotide polymorphism; SVS, small vessel stroke.

Table S3. Results of the heterogeneity tests conducted in the MR analysis.

Exposure	Outcome	Q	Q_df	*Q* value
IVW method				
Male infertility	AS	13.7349984	23	0.934209343
Male infertility	AIS	17.7557842	23	0.7705981
Male infertility	LAS	24.6801238	23	0.36698635
Male infertility	CES	23.8410356	22	0.35561693
Male infertility	SVS	32.8655793	23	0.08343765
MR Egger				
Male infertility	AS	13.7328876	22	0.910687688
Male infertility	AIS	17.56592	22	0.73139232
Male infertility	LAS	23.7672375	22	0.35954766
Male infertility	CES	17.864118	21	0.65758626
Male infertility	SVS	32.6201301	22	0.06742103

AIS, any ischemic stroke; AS, all stroke; CES, cardioembolic stroke; IVW, inverse-variance weighted; LAS, large artery stroke; MR, Mendelian randomization; SE, standard error; SVS, small vessel stroke.

Table S4. Results of the pleiotropy tests conducted in the MR analysis.

Exposure	Outcome	Egger_intercept	SE	*p* value
Male infertility	AS	-0.0002938	0.0063942	0.96376941
Male infertility	AIS	0.0030095	0.0069067	0.66727521
Male infertility	LAS	0.0164611	0.0179073	0.36793807
Male infertility	CES	0.0333354	0.0136354	0.02340024
Male infertility	SVS	-0.0079519	0.0195445	0.68803907

AIS, any ischemic stroke; AS, all stroke; CES, cardioembolic stroke; LAS, large artery stroke; MR, Mendelian randomization; SE, standard error; SVS, small vessel stroke.

Table S5. Results of the leave-one-out sensitivity analyses for all stroke.

Exposure	Outcome	SNP	Beta	SE	*p* value
Male infertility	AS	rs10054423	-0.0464328	0.03237618	0.15152514
Male infertility	AS	rs10458884	-0.0442431	0.0325698	0.17433366
Male infertility	AS	rs10503673	-0.0447514	0.03284386	0.17302419
Male infertility	AS	rs11073495	-0.0405229	0.03240684	0.21113759
Male infertility	AS	rs114845433	-0.0435923	0.03227315	0.17678244
Male infertility	AS	rs115338272	-0.0422848	0.03276949	0.19692155
Male infertility	AS	rs117557835	-0.0443049	0.03210029	0.16752483
Male infertility	AS	rs11886942	-0.042407	0.0318876	0.18355413
Male infertility	AS	rs12420380	-0.0394349	0.03193354	0.21686524
Male infertility	AS	rs13028479	-0.053965	0.03253413	0.09717246
Male infertility	AS	rs1424035	-0.0369184	0.03368383	0.2730672
Male infertility	AS	rs3020331	-0.0403202	0.03255924	0.2155811
Male infertility	AS	rs3816838	-0.0293446	0.0326688	0.36905445
Male infertility	AS	rs3986292	-0.0400109	0.03234476	0.21608233
Male infertility	AS	rs55938609	-0.0330232	0.03283051	0.31447824
Male infertility	AS	rs58415480	-0.0458496	0.03253301	0.15873861
Male infertility	AS	rs58924196	-0.0421531	0.03210537	0.18919598
Male infertility	AS	rs6025	-0.0384947	0.03266461	0.23860451
Male infertility	AS	rs6835215	-0.0481633	0.03286709	0.14281273
Male infertility	AS	rs73230212	-0.0429114	0.0328486	0.19143723
Male infertility	AS	rs73527055	-0.03282	0.0325177	0.31283196
Male infertility	AS	rs76071455	-0.0431056	0.03215656	0.18008515
Male infertility	AS	rs79909005	-0.0405123	0.03216371	0.20782612
Male infertility	AS	rs8032552	-0.0499076	0.03242807	0.12379827
Male infertility	AS	All	-0.0419154	0.03181218	0.18764151

AS, all stroke; SE, standard error; SNP, single nucleotide polymorphism.

Table S6. Results of the leave-one-out sensitivity analyses for any ischemic stroke.

Exposure	Outcome	SNP	Beta	SE	*p* value
Male infertility	AIS	rs10054423	-0.0318725	0.03488227	0.36086648
Male infertility	AIS	rs10458884	-0.0268221	0.0351201	0.44503164
Male infertility	AIS	rs10503673	-0.0349472	0.03539222	0.32343382
Male infertility	AIS	rs11073495	-0.027624	0.03495881	0.42941778
Male infertility	AIS	rs114845433	-0.0307999	0.03476798	0.37568749
Male infertility	AIS	rs115338272	-0.0261421	0.03532295	0.4592468
Male infertility	AIS	rs117557835	-0.0293894	0.03458551	0.39545835
Male infertility	AIS	rs11886942	-0.0281981	0.03437676	0.41206408
Male infertility	AIS	rs12420380	-0.0242326	0.03443919	0.48166113
Male infertility	AIS	rs13028479	-0.0385581	0.03509999	0.271977
Male infertility	AIS	rs1424035	-0.0246823	0.03627114	0.49619178
Male infertility	AIS	rs3020331	-0.027052	0.03510865	0.44098936
Male infertility	AIS	rs3816838	-0.0133436	0.03521002	0.70470911
Male infertility	AIS	rs3986292	-0.0311939	0.03487962	0.37114539
Male infertility	AIS	rs55938609	-0.0224314	0.03539801	0.5262825
Male infertility	AIS	rs58415480	-0.0326192	0.03507593	0.35239226
Male infertility	AIS	rs58924196	-0.0290435	0.03461523	0.40144766
Male infertility	AIS	rs6025	-0.0182357	0.0351877	0.60429063
Male infertility	AIS	rs6835215	-0.0342252	0.03542514	0.33398084
Male infertility	AIS	rs73230212	-0.0298242	0.03551618	0.40105694
Male infertility	AIS	rs73527055	-0.0187772	0.03506708	0.59232846
Male infertility	AIS	rs76071455	-0.0282336	0.03466853	0.41542311
Male infertility	AIS	rs79909005	-0.0221108	0.03469824	0.52397428
Male infertility	AIS	rs8032552	-0.0417871	0.03496967	0.23210507
Male infertility	AIS	All	-0.0280157	0.0342987	0.41403386

AIS, any ischemic stroke; SE, standard error; SNP, single nucleotide polymorphism.

Table S7. Results of the leave-one-out sensitivity analyses for large artery stroke.

Exposure	Outcome	SNP	Beta	SE	*p* value
Male infertility	LAS	rs10054423	-0.0424152	0.0913862	0.64255381
Male infertility	LAS	rs10458884	-0.0493439	0.0932762	0.59679972
Male infertility	LAS	rs10503673	-0.0636158	0.09437897	0.50028217
Male infertility	LAS	rs11073495	-0.0592688	0.09321028	0.52486663
Male infertility	LAS	rs114845433	-0.0687878	0.09113005	0.45035005
Male infertility	LAS	rs115338272	-0.0731279	0.09296982	0.43152973
Male infertility	LAS	rs117557835	-0.056807	0.09206022	0.53719257
Male infertility	LAS	rs11886942	-0.0622529	0.09060435	0.4920293
Male infertility	LAS	rs12420380	-0.056692	0.0917952	0.53684443
Male infertility	LAS	rs13028479	-0.094315	0.08838331	0.28592056
Male infertility	LAS	rs1424035	-0.0148861	0.09404317	0.87422863
Male infertility	LAS	rs3020331	-0.0850484	0.08979016	0.34354185
Male infertility	LAS	rs3816838	-0.0581978	0.09391843	0.53547982
Male infertility	LAS	rs3986292	-0.0314043	0.08770863	0.72030429
Male infertility	LAS	rs55938609	-0.0877897	0.09106731	0.33504125
Male infertility	LAS	rs58415480	-0.0698853	0.09274749	0.45114932
Male infertility	LAS	rs58924196	-0.0746394	0.0886347	0.39973142
Male infertility	LAS	rs6025	-0.07189	0.09299601	0.43949612
Male infertility	LAS	rs6835215	-0.0535516	0.09409052	0.56925482
Male infertility	LAS	rs73230212	-0.0259404	0.08973791	0.77252888
Male infertility	LAS	rs73527055	-0.0355907	0.09060713	0.69446561
Male infertility	LAS	rs76071455	-0.0536143	0.09204226	0.56023236
Male infertility	LAS	rs79909005	-0.0319286	0.08741844	0.71493289
Male infertility	LAS	rs8032552	-0.0656034	0.09298457	0.48048027
Male infertility	LAS	All	-0.0578801	0.08944332	0.51755782

LAS, large artery stroke; SE, standard error; SNP, single nucleotide polymorphism.

Table S8. Results of the leave-one-out sensitivity analyses for cardioembolic stroke.

Exposure	Outcome	SNP	Beta	SE	*p* value
Male infertility	CES	rs10054423	-0.063035	0.07291524	0.38731502
Male infertility	CES	rs10458884	-0.0543493	0.0738415	0.46171498
Male infertility	CES	rs10503673	-0.0841926	0.07061432	0.23314857
Male infertility	CES	rs11073495	-0.0696628	0.0720207	0.33341359
Male infertility	CES	rs114845433	-0.0572861	0.0729814	0.43248785
Male infertility	CES	rs115338272	-0.0498193	0.07396529	0.50059726
Male infertility	CES	rs117557835	-0.0472456	0.07088468	0.50508268
Male infertility	CES	rs12420380	-0.0512496	0.07157859	0.47399719
Male infertility	CES	rs13028479	-0.0441792	0.07287175	0.54434243
Male infertility	CES	rs1424035	-0.0154864	0.07129077	0.82803022
Male infertility	CES	rs3020331	-0.0505272	0.07366095	0.49274954
Male infertility	CES	rs3816838	-0.0611598	0.07393928	0.40814519
Male infertility	CES	rs3986292	-0.0699037	0.07155626	0.32861567
Male infertility	CES	rs55938609	-0.0290778	0.07049574	0.67999027
Male infertility	CES	rs58415480	-0.0661145	0.07299655	0.36508332
Male infertility	CES	rs58924196	-0.0638426	0.0716842	0.37313895
Male infertility	CES	rs6025	-0.0335983	0.06914479	0.62702965
Male infertility	CES	rs6835215	-0.0559127	0.07443085	0.45253014
Male infertility	CES	rs73230212	-0.0644181	0.07409062	0.38460096
Male infertility	CES	rs73527055	-0.0737081	0.0715784	0.30312546
Male infertility	CES	rs76071455	-0.0517367	0.07257029	0.47589634
Male infertility	CES	rs79909005	-0.0473853	0.07184456	0.50954085
Male infertility	CES	rs8032552	-0.0882668	0.06901162	0.20089247
Male infertility	CES	All	-0.0562683	0.07040499	0.42416915

CES, cardioembolic stroke; SE, standard error; SNP, single nucleotide polymorphism.

Table S9. Results of the leave-one-out sensitivity analyses for small vessel stroke.

Exposure	Outcome	SNP	Beta	SE	*p* value
Male infertility	SVS	rs3816838	-0.1431515	0.08907242	0.10802464
Male infertility	SVS	rs10503673	-0.1668213	0.09848026	0.09027346
Male infertility	SVS	rs73527055	-0.1661588	0.09611539	0.08385505
Male infertility	SVS	rs6025	-0.1729344	0.09801244	0.07766257
Male infertility	SVS	rs115338272	-0.1816007	0.10042481	0.07055599
Male infertility	SVS	rs114845433	-0.1779701	0.09800278	0.06937503
Male infertility	SVS	rs3020331	-0.1830241	0.09985932	0.06682944
Male infertility	SVS	rs58415480	-0.1861354	0.0999077	0.06245186
Male infertility	SVS	rs6835215	-0.1942851	0.10151824	0.05564638
Male infertility	SVS	rs79909005	-0.1892789	0.09883971	0.05549146
Male infertility	SVS	rs11073495	-0.1926951	0.09990522	0.0537583
Male infertility	SVS	rs12420380	-0.1762168	0.09065956	0.05192923
Male infertility	SVS	rs73230212	-0.2011983	0.10081355	0.04596151
Male infertility	SVS	rs10458884	-0.1998062	0.10011103	0.0459507
Male infertility	SVS	rs11886942	-0.195809	0.09784598	0.04537129
Male infertility	SVS	rs117557835	-0.1973849	0.09840915	0.04488211
Male infertility	SVS	rs76071455	-0.2040476	0.09804512	0.03741925
Male infertility	SVS	rs13028479	-0.2075877	0.09895996	0.03593217
Male infertility	SVS	rs55938609	-0.2119723	0.09991262	0.03387329
Male infertility	SVS	rs58924196	-0.2063845	0.09655318	0.0325556
Male infertility	SVS	rs10054423	-0.2102769	0.09760336	0.03120862
Male infertility	SVS	rs3986292	-0.2193969	0.09492565	0.02081907
Male infertility	SVS	rs1424035	-0.2331218	0.10042332	0.02026576
Male infertility	SVS	rs8032552	-0.2237864	0.09439116	0.01774769
Male infertility	SVS	All	-0.1933135	0.09582416	0.04365611

SE, standard error; SNP, single nucleotide polymorphism; SVS, small vessel stroke.

Table S10. The full list of MEGASTROKE authors.

Rainer Malik [1], Ganesh Chauhan [2], Matthew Traylor [3], Muralidharan Sargurupremraj [4,5], Yukinori Okada [6,7,8], Aniket Mishra [4,5], Loes Rutten-Jacobs [3], Anne-Katrin Giese [9], Sander W van der Laan [10], Solveig Gretarsdottir [11], Christopher D Anderson [12,13,14,14], Michael Chong [15], Hieab HH Adams [16,17], Tetsuro Ago [18], Peter Almgren [19], Philippe Amouyel [20,21], Hakan Ay [22,13], Traci M Bartz [23], Oscar R Benavente [24], Steve Bevan [25], Giorgio B Boncoraglio [26], Robert D Brown, Jr. [27], Adam S Butterworth [28,29], Caty Carrera [30,31], Cara L Carty [32,33], Daniel I Chasman [34,35], Wei-Min Chen [36], John W Cole [37], Adolfo Correa [38], Ioana Cotlarciuc [39], Carlos Cruchaga [40,41], John Danesh [28,42,43,44], Paul IW de Bakker [45,46], Anita L DeStefano [47,48], Marcel den Hoed [49], Qing Duan [50], Stefan T Engelter [51,52], Guido J Falcone [53,54], Rebecca F Gottesman [55], Raji P Grewal [56], Vilmundur Gudnason [57,58], Stefan Gustafsson [59], Jeffrey Haessler [60], Tamara B Harris [61], Ahamad Hassan [62], Aki S Havulinna [63,64], Susan R Heckbert [65], Elizabeth G Holliday [66,67], George Howard [68], Fang-Chi Hsu [69], Hyacinth I Hyacinth [70], M Arfan Ikram [16], Erik Ingelsson [71,72], Marguerite R Irvin [73], Xueqiu Jian [74], Jordi Jiménez-Conde [75], Julie A Johnson [76,77], J Wouter Jukema [78], Masahiro Kanai [6,7,79], Keith L Keene [80,81], Brett M Kissela [82], Dawn O Kleindorfer [82], Charles Kooperberg [60], Michiaki Kubo [83], Leslie A Lange [84], Carl D Langefeld [85], Claudia Langenberg [86], Lenore J Launer [87], Jin-Moo Lee [88], Robin Lemmens [89,90], Didier Leys [91], Cathryn M Lewis [92,93], Wei-Yu Lin [28,94], Arne G Lindgren [95,96], Erik Lorentzen [97], Patrik K Magnusson [98], Jane Maguire [99], Ani Manichaikul [36], Patrick F McArdle [100], James F Meschia [101], Braxton D Mitchell [100,102], Thomas H Mosley [103,104], Michael A Nalls [105,106], Toshiharu Ninomiya [107], Martin J O'Donnell [15,108], Bruce M Psaty [109,110,111,112], Sara L Pulit [113,45], Kristiina Rannikmäe [114,115], Alexander P Reiner [65,116], Kathryn M Rexrode [117], Kenneth Rice [118], Stephen S Rich [36], Paul M Ridker [34,35], Natalia S Rost [9,13], Peter M Rothwell [119], Jerome I Rotter [120,121], Tatjana Rundek [122], Ralph L Sacco [122], Saori Sakaue [7,123], Michele M Sale [124], Veikko Salomaa [63], Bishwa R Sapkota [125], Reinhold Schmidt [126], Carsten O Schmidt [127], Ulf Schminke [128], Pankaj Sharma [39], Agnieszka Slowik [129], Cathie LM Sudlow [114,115], Christian Tanislav [130], Turgut Tatlisumak [131,132], Kent D Taylor [120,121], Vincent NS Thijs [133,134], Gudmar Thorleifsson [11], Unnur Thorsteinsdottir [11], Steffen Tiedt [1], Stella Trompet [135], Christophe Tzourio [5,136,137], Cornelia M van Duijn [138,139], Matthew Walters [140], Nicholas J Wareham [86], Sylvia Wassertheil-Smoller [141], James G Wilson [142], Kerri L Wiggins [109], Qiong Yang [47], Salim Yusuf [15], Najaf Amin [16], Hugo S Aparicio [185,48], Donna K Arnett [186], John Attia [187], Alexa S Beiser [47,48], Claudine Berr [188], Julie E Buring [34,35], Mariana Bustamante [189], Valeria Caso [190], Yu-Ching Cheng [191], Seung Hoan Choi [192,48], Ayesha Chowhan [185,48], Natalia Cullell [31], Jean-François Dartigues [193,194], Hossein Delavaran [95,96], Pilar Delgado [195], Marcus Dörr [196,197], Gunnar Engström [19], Ian Ford [198], Wander S Gurpreet [199], Anders Hamsten [200,201], Laura Heitsch [202], Atsushi Hozawa [203], Laura Ibanez [204], Andreea Ilinca [95,96], Martin Ingelsson [205], Motoki Iwasaki [206], Rebecca D Jackson [207], Katarina Jood [208], Pekka Jousilahti [63], Sara Kaffashian [4,5], Lalit Kalra [209], Masahiro Kamouchi [210], Takanari Kitazono [211], Olafur Kjartansson [212], Manja Kloss [213], Peter J Koudstaal [214], Jerzy Krupinski [215], Daniel L Labovitz [216], Cathy C Laurie [118], Christopher R Levi [217], Linxin Li [218], Lars Lind [219], Cecilia M Lindgren [220,221], Vasileios Lioutas [222,48], Yong Mei Liu [223], Oscar L Lopez [224], Hirata Makoto [225], Nicolas Martinez-Majander [172], Koichi Matsuda [225], Naoko Minegishi [203], Joan Montaner [226], Andrew P Morris [227,228], Elena Muiño [31], Martina Müller-Nurasyid [229,230,231], Bo Norrving [95,96], Soichi Ogishima [203], Eugenio A Parati [232], Leema Reddy Peddareddygari [56], Nancy L Pedersen [98,233], Joanna Pera [129], Markus Perola [63,234], Alessandro Pezzini [235], Silvana Pileggi [236], Raquel Rabionet [237], Iolanda Riba-Llena [30], Marta Ribasés [238], Jose R Romero [185,48], Jaume Roquer [239,240], Anthony G Rudd [241,242],

Antti-Pekka Sarin [243,244], Ralhan Sarju [199], Chloe Sarnowski [47,48], Makoto Sasaki [245], Claudia L Satizabal [185,48], Mamoru Satoh [245], Naveed Sattar [246], Norie Sawada [206], Gerli Sibolt [172], Ásgeir Sigurdsson [247], Albert Smith [248], Kenji Sobue [245], Carolina Soriano-Tárraga [240], Tara Stanne [249], O Colin Stine [250], David J Stott [251], Konstantin Strauch [229,252], Takako Takai [203], Hideo Tanaka [253,254], Kozo Tanno [245], Alexander Teumer [255], Liisa Tomppo [172], Nuria P Torres-Aguila [31], Emmanuel Touze [256,257], Shoichiro Tsugane [206], Andre G Uitterlinden [258], Einar M Valdimarsson [259], Sven J van der Lee [16], Henry Völzke [255], Kenji Wakai [253], David Weir [260], Stephen R Williams [261], Charles DA Wolfe [241,242], Quenna Wong [118], Huichun Xu [191], Taiki Yamaji [206], Dharambir K Sanghera [125,169,170], Olle Melander [19], Christina Jern [171], Daniel Strbian [172,173], Israel Fernandez-Cadenas [31,30], W T Longstreth, Jr [174,65], Arndt Rolfs [175], Jun Hata [107], Daniel Woo [82], Jonathan Rosand [12,13,14], Guillaume Pare [15], Jemma C Hopewell [176], Danish Saleheen [177], Kari Stefansson [11,178], Bradford B Worrall [179], Steven J Kittner [37], Sudha Seshadri [180,48], Myriam Fornage [74,181], Hugh S Markus [3], Joanna MM Howson [28], Yoichiro Kamatani [6,182], Stephanie Debette [4,5], Martin Dichgans [1,183,184]

1 Institute for Stroke and Dementia Research (ISD), University Hospital, LMU Munich, Munich, Germany
2 Centre for Brain Research, Indian Institute of Science, Bangalore, India
3 Stroke Research Group, Division of Clinical Neurosciences, University of Cambridge, UK
4 INSERM U1219 Bordeaux Population Health Research Center, Bordeaux, France
5 University of Bordeaux, Bordeaux, France
6 Laboratory for Statistical Analysis, RIKEN Center for Integrative Medical Sciences, Yokohama, Japan
7 Department of Statistical Genetics, Osaka University Graduate School of Medicine, Osaka, Japan
8 Laboratory of Statistical Immunology, Immunology Frontier Research Center (WPI-IFReC), Osaka University, Suita, Japan.
9 Department of Neurology, Massachusetts General Hospital, Harvard Medical School, Boston, MA, USA
10 Laboratory of Experimental Cardiology, Division of Heart and Lungs, University Medical Center Utrecht, University of Utrecht, Utrecht,Netherlands
11 deCODE genetics/AMGEN inc, Reykjavik, Iceland
12 Center for Genomic Medicine, Massachusetts General Hospital (MGH), Boston, MA, USA
13 J. Philip Kistler Stroke Research Center, Department of Neurology, MGH, Boston, MA, USA
14 Program in Medical and Population Genetics, Broad Institute, Cambridge, MA, USA
15 Population Health Research Institute, McMaster University, Hamilton, Canada
16 Department of Epidemiology, Erasmus University Medical Center, Rotterdam, Netherlands
17 Department of Radiology and Nuclear Medicine, Erasmus University Medical Center, Rotterdam, Netherlands
18 Department of Medicine and Clinical Science, Graduate School of Medical Sciences, Kyushu University, Fukuoka, Japan
19 Department of Clinical Sciences, Lund University, Malmö, Sweden
20 Univ. Lille, Inserm, Institut Pasteur de Lille, LabEx DISTALZ-UMR1167, Risk factors and molecular determinants of aging-related diseases, F-59000 Lille, France
21 Centre Hosp. Univ Lille, Epidemiology and Public Health Department, F-59000 Lille, France

22 AA Martinos Center for Biomedical Imaging, Department of Radiology, Massachusetts General Hospital, Harvard Medical School, Boston, MA, USA
23 Cardiovascular Health Research Unit, Departments of Biostatistics and Medicine, University of Washington, Seattle, WA, USA
24 Division of Neurology, Faculty of Medicine, Brain Research Center, University of British Columbia, Vancouver, Canada
25 School of Life Science, University of Lincoln, Lincoln, UK
26 Department of Cerebrovascular Diseases, Fondazione IRCCS Istituto Neurologico "Carlo Besta", Milano, Italy
27 Department of Neurology, Mayo Clinic Rochester, Rochester, MN, USA
28 MRC/BHF Cardiovascular Epidemiology Unit, Department of Public Health and Primary Care, University of Cambridge, Cambridge, UK
29 The National Institute for Health Research Blood and Transplant Research Unit in Donor Health and Genomics, University of Cambridge, UK
30 Neurovascular Research Laboratory, Vall d'Hebron Institut of Research, Neurology and Medicine Departments-Universitat Autònoma de Barcelona, Vall d’Hebrón Hospital, Barcelona, Spain
31 Stroke Pharmacogenomics and Genetics, Fundacio Docència i Recerca MutuaTerrassa, Terrassa, Spain
32 Children's Research Institute, Children's National Medical Center, Washington, DC, USA
33 Center for Translational Science, George Washington University, Washington, DC, USA
34 Division of Preventive Medicine, Brigham and Women's Hospital, Boston, MA, USA
35 Harvard Medical School, Boston, MA, USA
36 Center for Public Health Genomics, Department of Public Health Sciences, University of Virginia, Charlottesville, VA, USA
37 Department of Neurology, University of Maryland School of Medicine and Baltimore VAMC, Baltimore, MD, USA
38 Departments of Medicine, Pediatrics and Population Health Science, University of Mississippi Medical Center, Jackson, MS, USA
39 Institute of Cardiovascular Research, Royal Holloway University of London, UK & Ashford and St Peters Hospital, Surrey UK
40 Department of Psychiatry,The Hope Center Program on Protein Aggregation and Neurodegeneration (HPAN),Washington University, School of Medicine, St. Louis, MO, USA
41 Department of Developmental Biology, Washington University School of Medicine, St. Louis, MO, USA
42 NIHR Blood and Transplant Research Unit in Donor Health and Genomics, Department of Public Health and Primary Care, University of Cambridge, Cambridge, UK
43 Wellcome Trust Sanger Institute, Wellcome Trust Genome Campus, Hinxton, Cambridge, UK
44 British Heart Foundation, Cambridge Centre of Excellence, Department of Medicine, University of Cambridge, Cambridge, UK
45 Department of Medical Genetics, University Medical Center Utrecht, Utrecht, Netherlands
46 Department of Epidemiology, Julius Center for Health Sciences and Primary Care, University Medical Center Utrecht, Utrecht, Netherlands
47 Boston University School of Public Health, Boston, MA, USA
48 Framingham Heart Study, Framingham, MA, USA

49 Department of Immunology, Genetics and Pathology and Science for Life Laboratory, Uppsala University, Uppsala, Sweden
50 Department of Genetics, University of North Carolina, Chapel Hill, NC, USA
51 Department of Neurology and Stroke Center, Basel University Hospital, Switzerland
52 Neurorehabilitation Unit, University and University Center for Medicine of Aging and Rehabilitation Basel, Felix Platter Hospital, Basel, Switzerland
53 Department of Neurology, Yale University School of Medicine, New Haven, CT, USA
54 Program in Medical and Population Genetics, The Broad Institute of Harvard and MIT, Cambridge, MA, USA
55 Department of Neurology, Johns Hopkins University School of Medicine, Baltimore, MD, USA
56 Neuroscience Institute, SF Medical Center, Trenton, NJ, USA
57 Icelandic Heart Association Research Institute, Kopavogur, Iceland
58 University of Iceland, Faculty of Medicine, Reykjavik, Iceland
59 Department of Medical Sciences, Molecular Epidemiology and Science for Life Laboratory, Uppsala University, Uppsala, Sweden
60 Division of Public Health Sciences, Fred Hutchinson Cancer Research Center, Seattle, WA, USA
61 Laboratory of Epidemiology and Population Science, National Institute on Aging, National Institutes of Health, Bethesda, MD, USA
62 Department of Neurology, Leeds General Infirmary, Leeds Teaching Hospitals NHS Trust, Leeds, UK
63 National Institute for Health and Welfare, Helsinki, Finland
64 FIMM - Institute for Molecular Medicine Finland, Helsinki, Finland
65 Department of Epidemiology, University of Washington, Seattle, WA, USA
66 Public Health Stream, Hunter Medical Research Institute, New Lambton, Australia
67 Faculty of Health and Medicine, University of Newcastle, Newcastle, Australia
68 School of Public Health, University of Alabama at Birmingham, Birmingham, AL, USA
69 Department of Biostatistical Sciences, Wake Forest School of Medicine, Winston-Salem, NC, USA
70 Aflac Cancer and Blood Disorder Center, Department of Pediatrics, Emory University School of Medicine, Atlanta, GA, USA
71 Department of Medicine, Division of Cardiovascular Medicine, Stanford University School of Medicine, CA, USA
72 Department of Medical Sciences, Molecular Epidemiology and Science for Life Laboratory, Uppsala University, Uppsala, Sweden
73 Epidemiology, School of Public Health, University of Alabama at Birmingham, USA
74 Brown Foundation Institute of Molecular Medicine, University of Texas Health Science Center at Houston, Houston, TX, USA
75 Neurovascular Research Group (NEUVAS), Neurology Department, Institut Hospital del Mar d'Investigació Mèdica, Universitat Autònoma de Barcelona, Barcelona, Spain
76 Department of Pharmacotherapy and Translational Research and Center for Pharmacogenomics, University of Florida, College of Pharmacy, Gainesville, FL, USA
77 Division of Cardiovascular Medicine, College of Medicine, University of Florida, Gainesville, FL, USA
78 Department of Cardiology, Leiden University Medical Center, Leiden, the Netherlands
79 Program in Bioinformatics and Integrative Genomics, Harvard Medical School, Boston, MA, USA

80 Department of Biology, East Carolina University, Greenville, NC, USA
81 Center for Health Disparities, East Carolina University, Greenville, NC, USA
82 University of Cincinnati College of Medicine, Cincinnati, OH, USA
83 RIKEN Center for Integrative Medical Sciences, Yokohama, Japan
84 Department of Medicine, University of Colorado Denver, Anschutz Medical Campus, Aurora, CO, USA
85 Center for Public Health Genomics and Department of Biostatistical Sciences, Wake Forest School of Medicine, Winston-Salem, NC, USA
86 MRC Epidemiology Unit, University of Cambridge School of Clinical Medicine, Institute of Metabolic Science, Cambridge Biomedical Campus, Cambridge, UK
87 Intramural Research Program, National Institute on Aging, National Institutes of Health, Bethesda, MD, USA
88 Department of Neurology, Radiology, and Biomedical Engineering, Washington University School of Medicine, St. Louis, MO, USA
89 KU Leuven – University of Leuven, Department of Neurosciences, Experimental Neurology, Leuven, Belgium
90 VIB Center for Brain & Disease Research, University Hospitals Leuven, Department of Neurology, Leuven, Belgium
91 Univ.-Lille, INSERM U 1171. CHU Lille. Lille, France
92 Department of Medical and Molecular Genetics, King's College London, London, UK
93 SGDP Centre, Institute of Psychiatry, Psychology & Neuroscience, King's College London, London, UK
94 Northern Institute for Cancer Research, Paul O'Gorman Building, Newcastle University, Newcastle, UK
95 Department of Clinical Sciences Lund, Neurology, Lund University, Lund, Sweden
96 Department of Neurology and Rehabilitation Medicine, Skåne University Hospital, Lund, Sweden
97 Bioinformatics Core Facility, University of Gothenburg, Gothenburg, Sweden
98 Department of Medical Epidemiology and Biostatistics, Karolinska Institute, Stockholm, Sweden
99 University of Technology Sydney, Faculty of Health, Ultimo, Australia
100 Department of Medicine, University of Maryland School of Medicine, MD, USA
101 Department of Neurology, Mayo Clinic, Jacksonville, FL, USA
102 Geriatrics Research and Education Clinical Center, Baltimore Veterans Administration Medical Center, Baltimore, MD, USA
103 Division of Geriatrics, School of Medicine, University of Mississippi Medical Center, Jackson, MS, USA
104 Memory Impairment and Neurodegenerative Dementia Center, University of Mississippi Medical Center, Jackson, MS, USA
105 Laboratory of Neurogenetics, National Institute on Aging, National institutes of Health, Bethesda, MD, USA
106 Data Tecnica International, Glen Echo MD, USA
107 Department of Epidemiology and Public Health, Graduate School of Medical Sciences, Kyushu University, Fukuoka, Japan
108 Clinical Research Facility, Department of Medicine, NUI Galway, Galway, Ireland

109 Cardiovascular Health Research Unit, Department of Medicine, University of Washington, Seattle, WA, USA
110 Department of Epidemiology, University of Washington, Seattle, WA
111 Department of Health Services, University of Washington, Seattle, WA, USA
112 Kaiser Permanente Washington Health Research Institute, Seattle, WA, USA
113 Brain Center Rudolf Magnus, Department of Neurology, University Medical Center Utrecht, Utrecht, The Netherlands
114 Usher Institute of Population Health Sciences and Informatics, University of Edinburgh, Edinburgh, UK
115 Centre for Clinical Brain Sciences, University of Edinburgh, Edinburgh, UK
116 Fred Hutchinson Cancer Research Center, University of Washington, Seattle, WA, USA
117 Department of Medicine, Brigham and Women's Hospital, Boston, MA, USA
118 Department of Biostatistics, University of Washington, Seattle, WA, USA
119 Nuffield Department of Clinical Neurosciences, University of Oxford, UK
120 Institute for Translational Genomics and Population Sciences, Los Angeles Biomedical Research Institute at Harbor-UCLA Medical Center, Torrance, CA, USA
121 Division of Genomic Outcomes, Department of Pediatrics, Harbor-UCLA Medical Center, Torrance, CA, USA
122 Department of Neurology, Miller School of Medicine, University of Miami, Miami, FL, USA
123 Department of Allergy and Rheumatology, Graduate School of Medicine, the University of Tokyo, Tokyo, Japan
124 Center for Public Health Genomics, University of Virginia, Charlottesville, VA, USA
125 Department of Pediatrics, College of Medicine, University of Oklahoma Health Sciences Center, Oklahoma City, OK, USA
126 Department of Neurology, Medical University of Graz, Graz, Austria
127 University Medicine Greifswald, Institute for Community Medicine, SHIP-KEF, Greifswald, Germany
128 University Medicine Greifswald, Department of Neurology, Greifswald, Germany
129 Department of Neurology, Jagiellonian University, Krakow, Poland
130 Department of Neurology, Justus Liebig University, Giessen, Germany
131 Department of Clinical Neurosciences/Neurology, Institute of Neuroscience and Physiology, Sahlgrenska Academy at University of Gothenburg, Gothenburg, Sweden
132 Sahlgrenska University Hospital, Gothenburg, Sweden
133 Stroke Division, Florey Institute of Neuroscience and Mental Health, University of Melbourne, Heidelberg, Australia
134 Austin Health, Department of Neurology, Heidelberg, Australia
135 Department of Internal Medicine, Section Gerontology and Geriatrics, Leiden University Medical Center, Leiden, the Netherlands
136 INSERM U1219, Bordeaux, France
137 Department of Public Health, Bordeaux University Hospital, Bordeaux, France
138 Genetic Epidemiology Unit, Department of Epidemiology, Erasmus University Medical Center Rotterdam, Netherlands
139 Center for Medical Systems Biology, Leiden, Netherlands
140 School of Medicine, Dentistry and Nursing at the University of Glasgow, Glasgow, UK

141 Department of Epidemiology and Population Health, Albert Einstein College of Medicine, NY, USA
142 Department of Physiology and Biophysics, University of Mississippi Medical Center, Jackson, MS, USA
143 A full list of members and affiliations appears in the Supplementary Note
144 Department of Human Genetics, McGill University, Montreal, Canada
145 Department of Pathophysiology, Institute of Biomedicine and Translation Medicine, University of Tartu, Tartu, Estonia
146 Department of Cardiac Surgery, Tartu University Hospital, Tartu, Estonia
147 Clinical Gene Networks AB,Stockholm, Sweden
148 Department of Genetics and Genomic Sciences, The Icahn Institute for Genomics and Multiscale Biology Icahn School of Medicine at Mount Sinai, New York, NY, USA
149 Department of Pathophysiology, Institute of Biomedicine and Translation Medicine, University of Tartu, Biomeedikum, Tartu, Estonia
150 Integrated Cardio Metabolic Centre, Department of Medicine, Karolinska Institutet, Karolinska Universitetssjukhuset, Huddinge, Sweden.
151 Clinical Gene Networks AB, Stockholm, Sweden
152 Sorbonne Universités, UPMC Univ. Paris 06, INSERM, UMR_S 1166, Team Genomics & Pathophysiology of Cardiovascular Diseases, Paris, France
153 ICAN Institute for Cardiometabolism and Nutrition, Paris, France
154 Department of Biomedical Engineering, University of Virginia, Charlottesville, VA, USA
155 Group Health Research Institute, Group Health Cooperative, Seattle, WA, USA
156 Seattle Epidemiologic Research and Information Center, VA Office of Research and Development, Seattle, WA, USA
157 Cardiovascular Research Center, Massachusetts General Hospital, Boston, MA, USA
158 Department of Medical Research, Bærum Hospital, Vestre Viken Hospital Trust, Gjettum, Norway
159 Saw Swee Hock School of Public Health, National University of Singapore and National University Health System, Singapore
160 National Heart and Lung Institute, Imperial College London, London, UK
161 Department of Gene Diagnostics and Therapeutics, Research Institute, National Center for Global Health and Medicine, Tokyo, Japan
162 Department of Epidemiology, Tulane University School of Public Health and Tropical Medicine, New Orleans, LA, USA
163 Department of Cardiology,University Medical Center Groningen, University of Groningen, Netherlands
164 MRC-PHE Centre for Environment and Health, School of Public Health, Department of Epidemiology and Biostatistics, Imperial College London, London, UK
165 Department of Epidemiology and Biostatistics, Imperial College London, London, UK
166 Department of Cardiology, Ealing Hospital NHS Trust, Southall, UK
167 National Heart, Lung and Blood Research Institute, Division of Intramural Research, Population Sciences Branch, Framingham, MA, USA
168 A full list of members and affiliations appears at the end of the manuscript
169 Department of Phamaceutical Sciences, Collge of Pharmacy, University of Oklahoma Health Sciences Center, Oklahoma City, OK, USA

170 Oklahoma Center for Neuroscience, Oklahoma City, OK, USA
171 Department of Pathology and Genetics, Institute of Biomedicine, The Sahlgrenska Academy at University of Gothenburg, Gothenburg, Sweden
172 Department of Neurology, Helsinki University Hospital, Helsinki, Finland
173 Clinical Neurosciences, Neurology, University of Helsinki, Helsinki, Finland
174 Department of Neurology, University of Washington, Seattle, WA, USA
175 Albrecht Kossel Institute, University Clinic of Rostock, Rostock, Germany
176 Clinical Trial Service Unit and Epidemiological Studies Unit, Nuffield Department of Population Health, University of Oxford, Oxford, UK
177 Department of Genetics, Perelman School of Medicine, University of Pennsylvania, PA, USA
178 Faculty of Medicine, University of Iceland, Reykjavik, Iceland
179 Departments of Neurology and Public Health Sciences, University of Virginia School of Medicine, Charlottesville, VA, USA
180 Department of Neurology, Boston University School of Medicine, Boston, MA, USA
181 Human Genetics Center, University of Texas Health Science Center at Houston, Houston, TX, USA
182 Center for Genomic Medicine, Kyoto University Graduate School of Medicine, Kyoto, Japan
183 Munich Cluster for Systems Neurology (SyNergy), Munich, Germany
184 German Center for Neurodegenerative Diseases (DZNE), Munich, Germany
185 Boston University School of Medicine, Boston, MA, USA
186 University of Kentucky College of Public Health, Lexington, KY, USA
187 University of Newcastle and Hunter Medical Research Institute, New Lambton, Australia
188 Univ. Montpellier, Inserm, U1061, Montpellier, France
189 Centre for Research in Environmental Epidemiology, Barcelona, Spain
190 Department of Neurology, Università degli Studi di Perugia, Umbria, Italy
191 Department of Medicine, University of Maryland School of Medicine, Baltimore, MD, USA
192 Broad Institute, Cambridge, MA, USA
193 Univ. Bordeaux, Inserm, Bordeaux Population Health Research Center, UMR 1219, Bordeaux, France
194 Bordeaux University Hospital, Department of Neurology, Memory Clinic, Bordeaux, France
195 Neurovascular Research Laboratory. Vall d'Hebron Institut of Research, Neurology and Medicine Departments-Universitat Autònoma de Barcelona. Vall d’Hebrón Hospital, Barcelona, Spain
196 University Medicine Greifswald, Department of Internal Medicine B, Greifswald, Germany
197 DZHK, Greifswald, Germany
198 Robertson Center for Biostatistics, University of Glasgow, Glasgow, UK
199 Hero DMC Heart Institute, Dayanand Medical College & Hospital, Ludhiana, India
200 Atherosclerosis Research Unit, Department of Medicine Solna, Karolinska Institutet, Stockholm, Sweden
201 Karolinska Institutet, Stockholm, Sweden
202 Division of Emergency Medicine, and Department of Neurology, Washington University School of Medicine, St. Louis, MO, USA
203 Tohoku Medical Megabank Organization, Sendai, Japan
204 Department of Psychiatry, Washington University School of Medicine, St. Louis, MO, USA

205 Department of Public Health and Caring Sciences / Geriatrics, Uppsala University, Uppsala, Sweden
206 Epidemiology and Prevention Group, Center for Public Health Sciences, National Cancer Center, Tokyo, Japan
207 Department of Internal Medicine and the Center for Clinical and Translational Science, The Ohio State University, Columbus, OH, USA
208 Institute of Neuroscience and Physiology, the Sahlgrenska Academy at University of Gothenburg, Goteborg, Sweden
209 Department of Basic and Clinical Neurosciences, King's College London, London, UK
210 Department of Health Care Administration and Management, Graduate School of Medical Sciences, Kyushu University, Japan
211 Department of Medicine and Clinical Science, Graduate School of Medical Sciences, Kyushu University, Japan
212 Landspitali National University Hospital, Departments of Neurology & Radiology, Reykjavik, Iceland
213 Department of Neurology, Heidelberg University Hospital, Germany
214 Department of Neurology, Erasmus University Medical Center
215 Hospital Universitari Mutua Terrassa, Terrassa (Barcelona), Spain
216 Albert Einstein College of Medicine, Montefiore Medical Center, New York, NY, USA
217 John Hunter Hospital, Hunter Medical Research Institute and University of Newcastle, Newcastle, NSW, Australia
218 Centre for Prevention of Stroke and Dementia, Nuffield Department of Clinical Neurosciences, University of Oxford, UK
219 Department of Medical Sciences, Uppsala University, Uppsala, Sweden
220 Genetic and Genomic Epidemiology Unit, Wellcome Trust Centre for Human Genetics, University of Oxford, Oxford, UK
221 The Wellcome Trust Centre for Human Genetics, Oxford, UK
222 Beth Israel Deaconess Medical Center, Boston, MA, USA
223 Wake Forest School of Medicine, Wake Forest, NC, USA
224 Department of Neurology, University of Pittsburgh, Pittsburgh, PA, USA
225 BioBank Japan, Laboratory of Clinical Sequencing, Department of Computational biology and medical Sciences, Graduate school of Frontier Sciences, The University of Tokyo, Tokyo, Japan
226 Neurovascular Research Laboratory, Vall d'Hebron Institut of Research, Neurology and Medicine Departments-Universitat Autònoma de Barcelona. Vall d’Hebrón Hospital, Barcelona, Spain
227 Department of Biostatistics, University of Liverpool, Liverpool, UK
228 Wellcome Trust Centre for Human Genetics, University of Oxford, Oxford, UK
229 Institute of Genetic Epidemiology, Helmholtz Zentrum München - German Research Center for Environmental Health, Neuherberg, Germany
230 Department of Medicine I, Ludwig-Maximilians-Universität, Munich, Germany
231 DZHK (German Centre for Cardiovascular Research), partner site Munich Heart Alliance, Munich, Germany
232 Department of Cerebrovascular Diseases, Fondazione IRCCS Istituto Neurologico “Carlo Besta”, Milano, Italy
233 Karolinska Institutet, MEB, Stockholm, Sweden

234 University of Tartu, Estonian Genome Center, Tartu, Estonia, Tartu, Estonia
235 Department of Clinical and Experimental Sciences, Neurology Clinic, University of Brescia, Italy
236 Translational Genomics Unit, Department of Oncology, IRCCS Istituto di Ricerche Farmacologiche Mario Negri, Milano, Italy
237 Department of Genetics, Microbiology and Statistics, University of Barcelona, Barcelona, Spain
238 Psychiatric Genetics Unit, Group of Psychiatry, Mental Health and Addictions, Vall d'Hebron Research Institute (VHIR), Universitat Autònoma de Barcelona, Biomedical Network Research Centre on Mental Health (CIBERSAM), Barcelona, Spain
239 Department of Neurology, IMIM-Hospital del Mar, and Universitat Autònoma de Barcelona, Spain
240 IMIM (Hospital del Mar Medical Research Institute), Barcelona, Spain
241 National Institute for Health Research Comprehensive Biomedical Research Centre, Guy's & St. Thomas' NHS Foundation Trust and King's College London, London, UK
242 Division of Health and Social Care Research, King's College London, London, UK
243 FIMM-Institute for Molecular Medicine Finland, Helsinki, Finland
244 THL-National Institute for Health and Welfare, Helsinki, Finland
245 Iwate Tohoku Medical Megabank Organization, Iwate Medical University, Iwate, Japan
246 BHF Glasgow Cardiovascular Research Centre, Faculty of Medicine, Glasgow, UK
247 deCODE Genetics/Amgen, Inc., Reykjavik, Iceland
248 Icelandic Heart Association, Reykjavik, Iceland
249 Institute of Biomedicine, the Sahlgrenska Academy at University of Gothenburg, Goteborg, Sweden
250 Department of Epidemiology, University of Maryland School of Medicine, Baltimore, MD, USA
251 Institute of Cardiovascular and Medical Sciences, Faculty of Medicine, University of Glasgow, Glasgow, UK
252 Chair of Genetic Epidemiology, IBE, Faculty of Medicine, LMU Munich, Germany
253 Division of Epidemiology and Prevention, Aichi Cancer Center Research Institute, Nagoya, Japan
254 Department of Epidemiology, Nagoya University Graduate School of Medicine, Nagoya, Japan
255 University Medicine Greifswald, Institute for Community Medicine, SHIP-KEF, Greifswald, Germany
256 Department of Neurology, Caen University Hospital, Caen, France
257 University of Caen Normandy, Caen, France
258 Department of Internal Medicine, Erasmus University Medical Center, Rotterdam, Netherlands
259 Landspitali University Hospital, Reykjavik, Iceland
260 Survey Research Center, University of Michigan, Ann Arbor, MI, USA
261 University of Virginia Department of Neurology, Charlottesville, VA, USA

Supplementary Figures

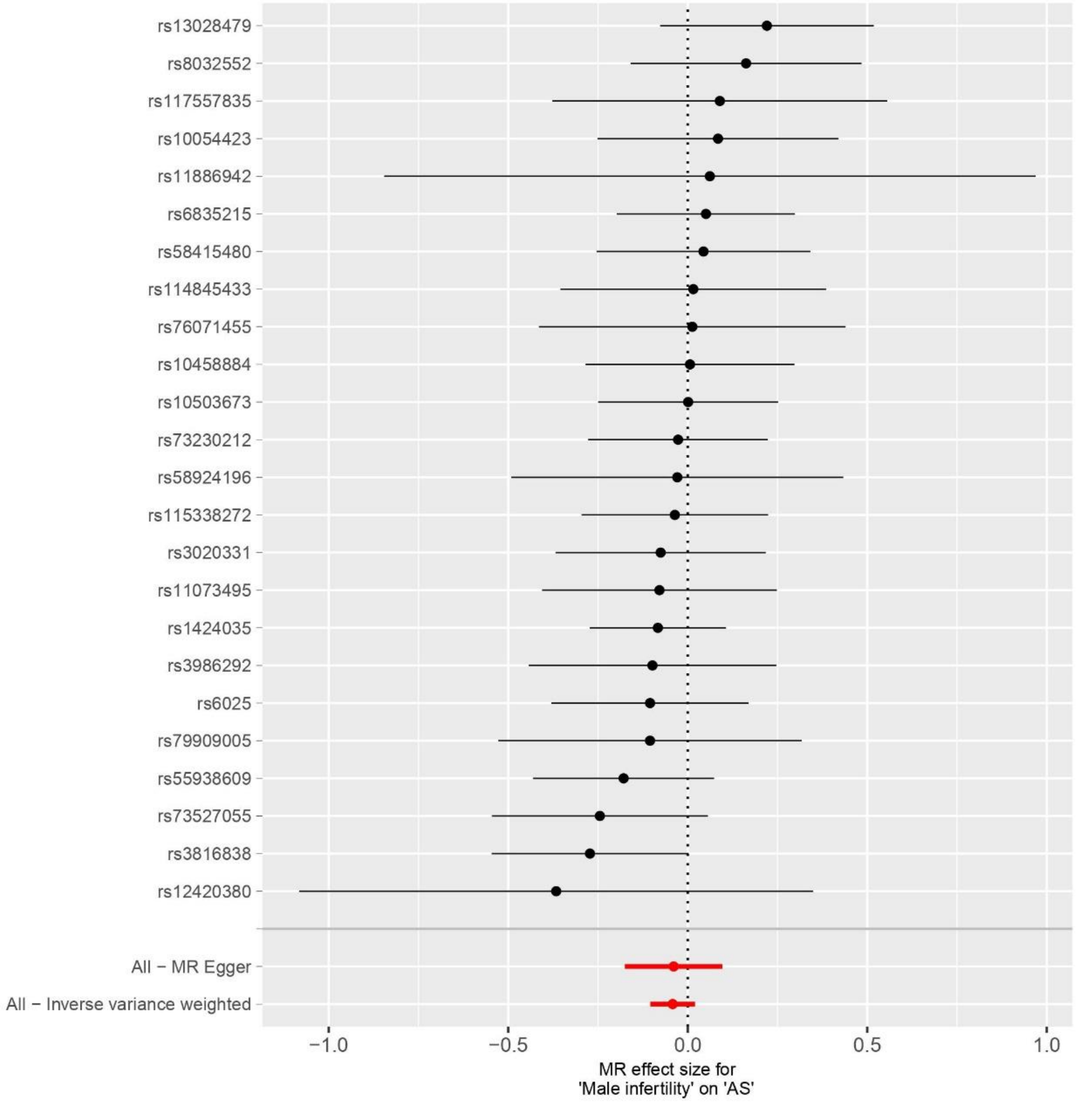

Figure S1. Forest plot of effect estimates for the causal relationship between male infertility and all stroke risk at each SNP level. AS, all stroke; MR, Mendelian randomization; SNP, single nucleotide polymorphism.

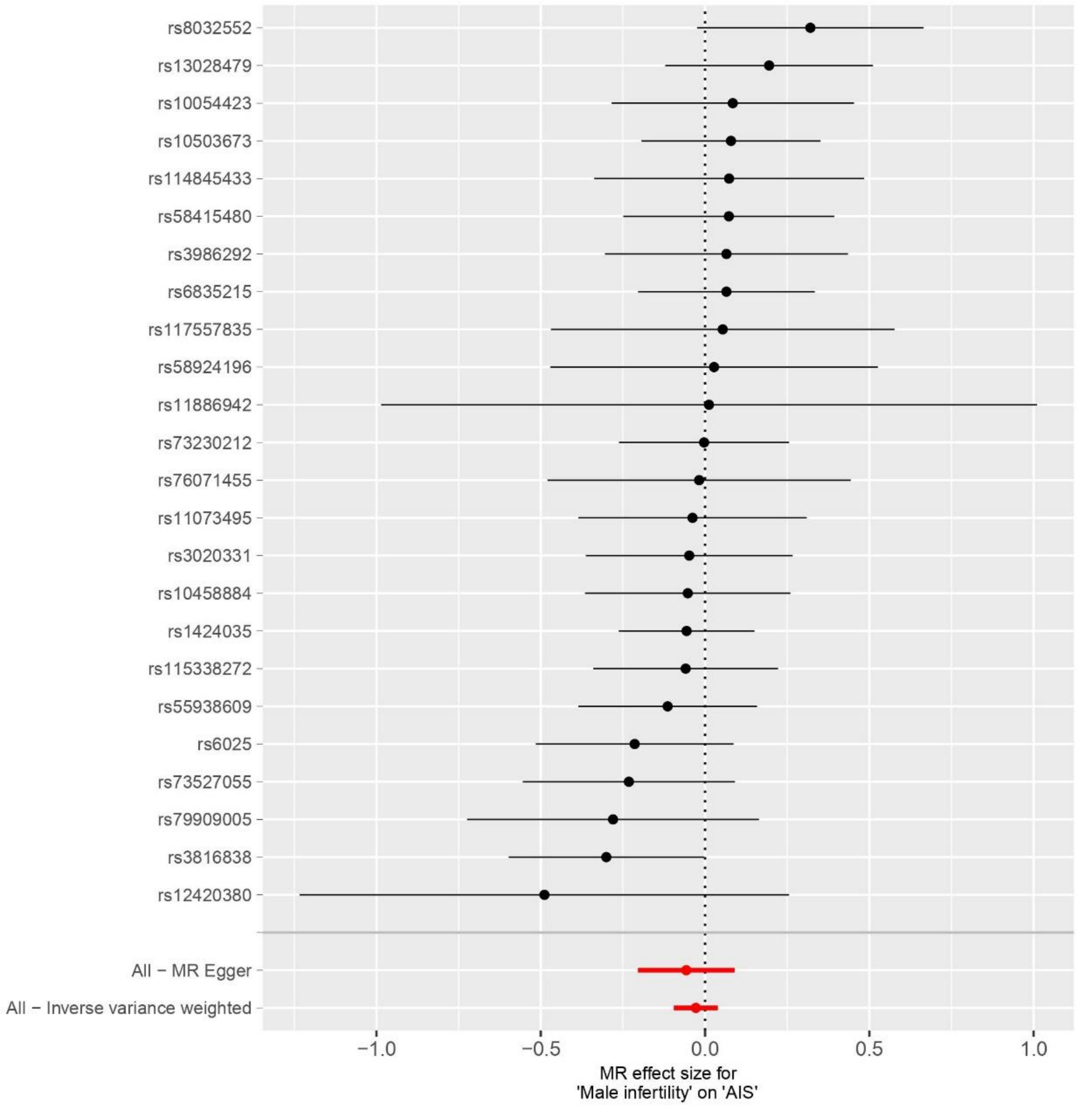

Figure S2. Forest plot of effect estimates for the causal relationship between male infertility and any ischemic stroke risk at each SNP level. AIS, any ischemic stroke; MR, Mendelian randomization; SNP, single nucleotide polymorphism.

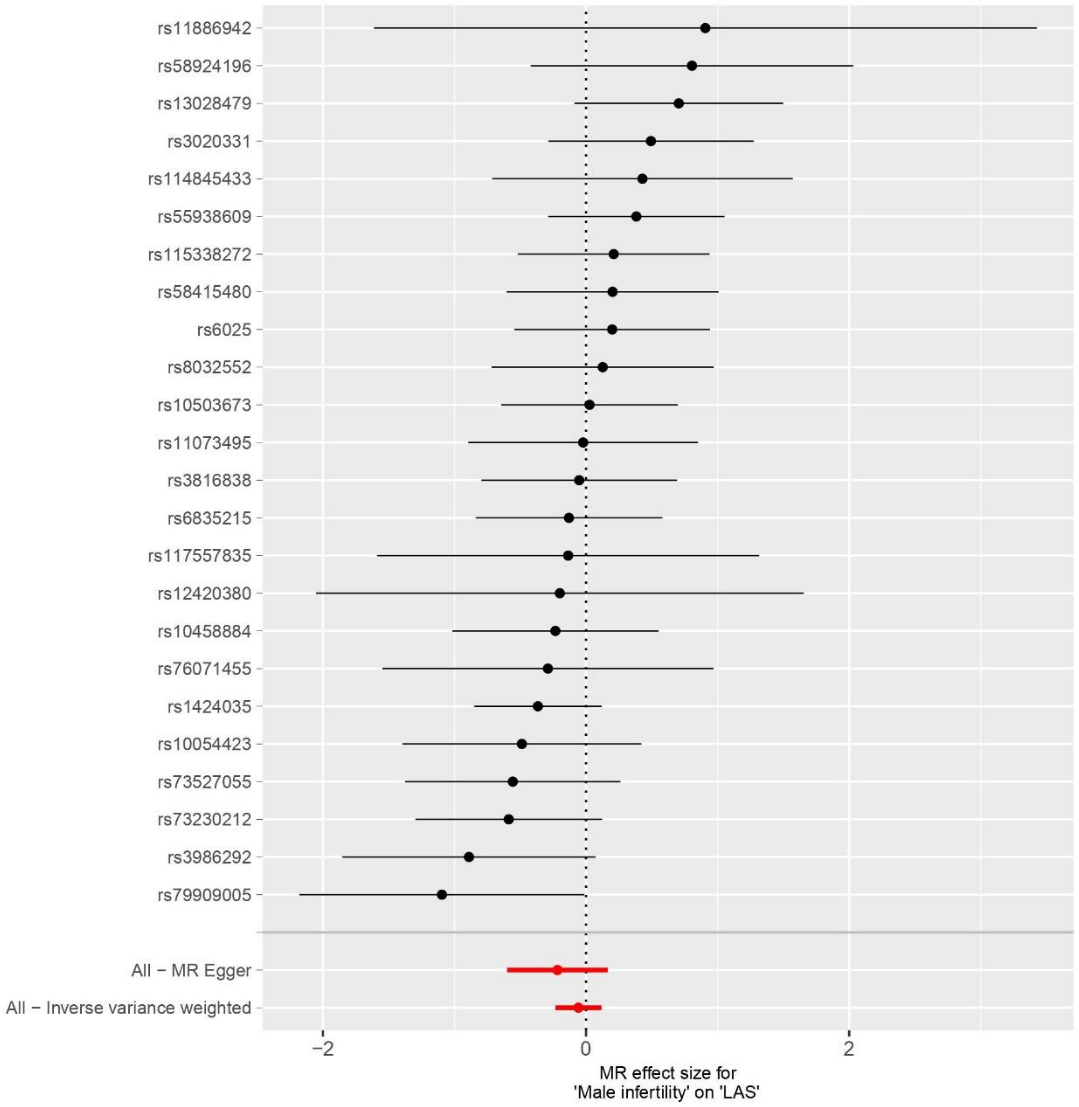

Figure S3. Forest plot of effect estimates for the causal relationship between male infertility and large artery stroke risk at each SNP level. LAS, large artery stroke; MR, Mendelian randomization; SNP, single nucleotide polymorphism.

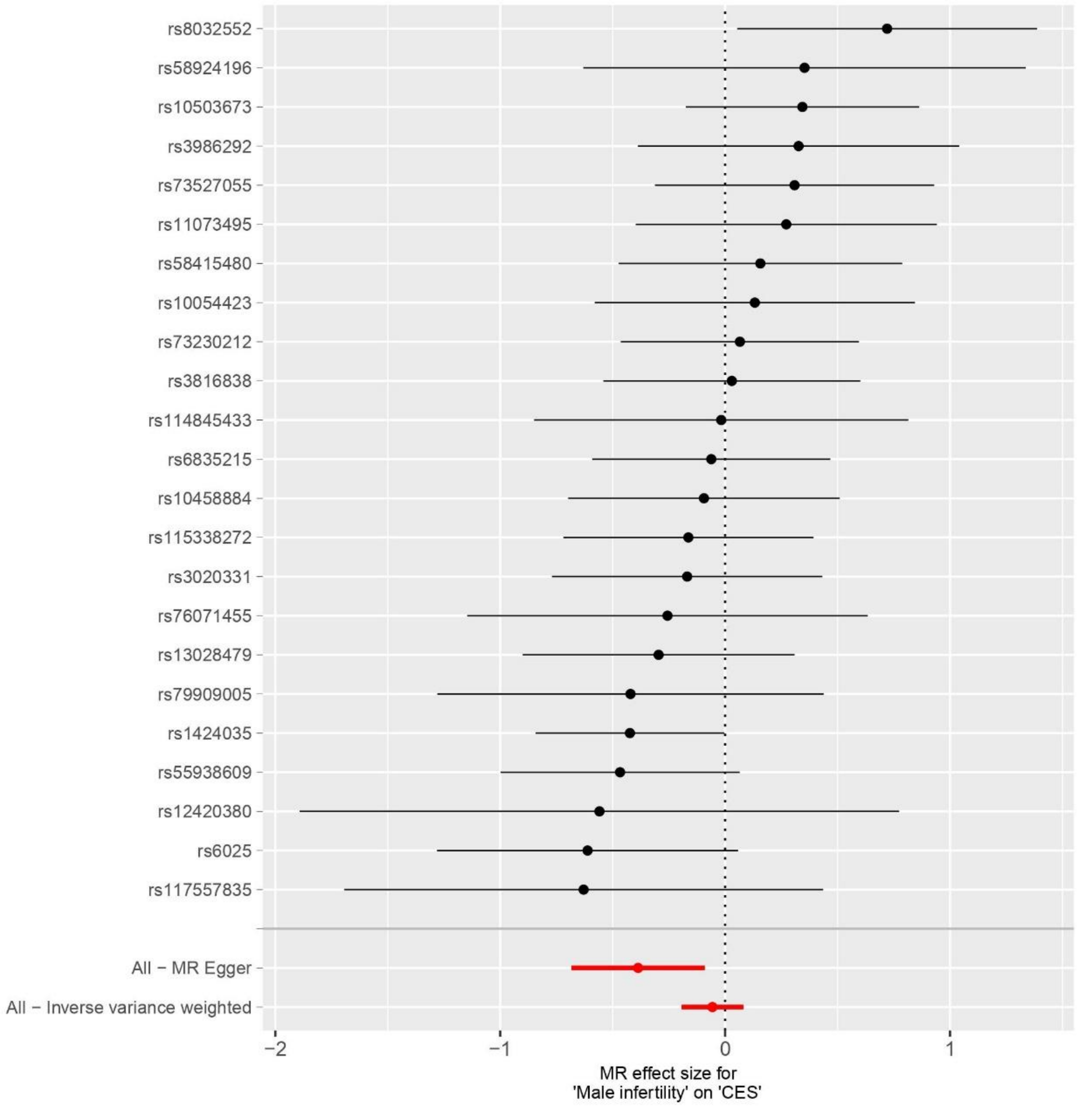

Figure S4. Forest plot of effect estimates for the causal relationship between male infertility and cardioembolic stroke risk at each SNP level. CES, cardioembolic stroke; MR, Mendelian randomization; SNP, single nucleotide polymorphism.

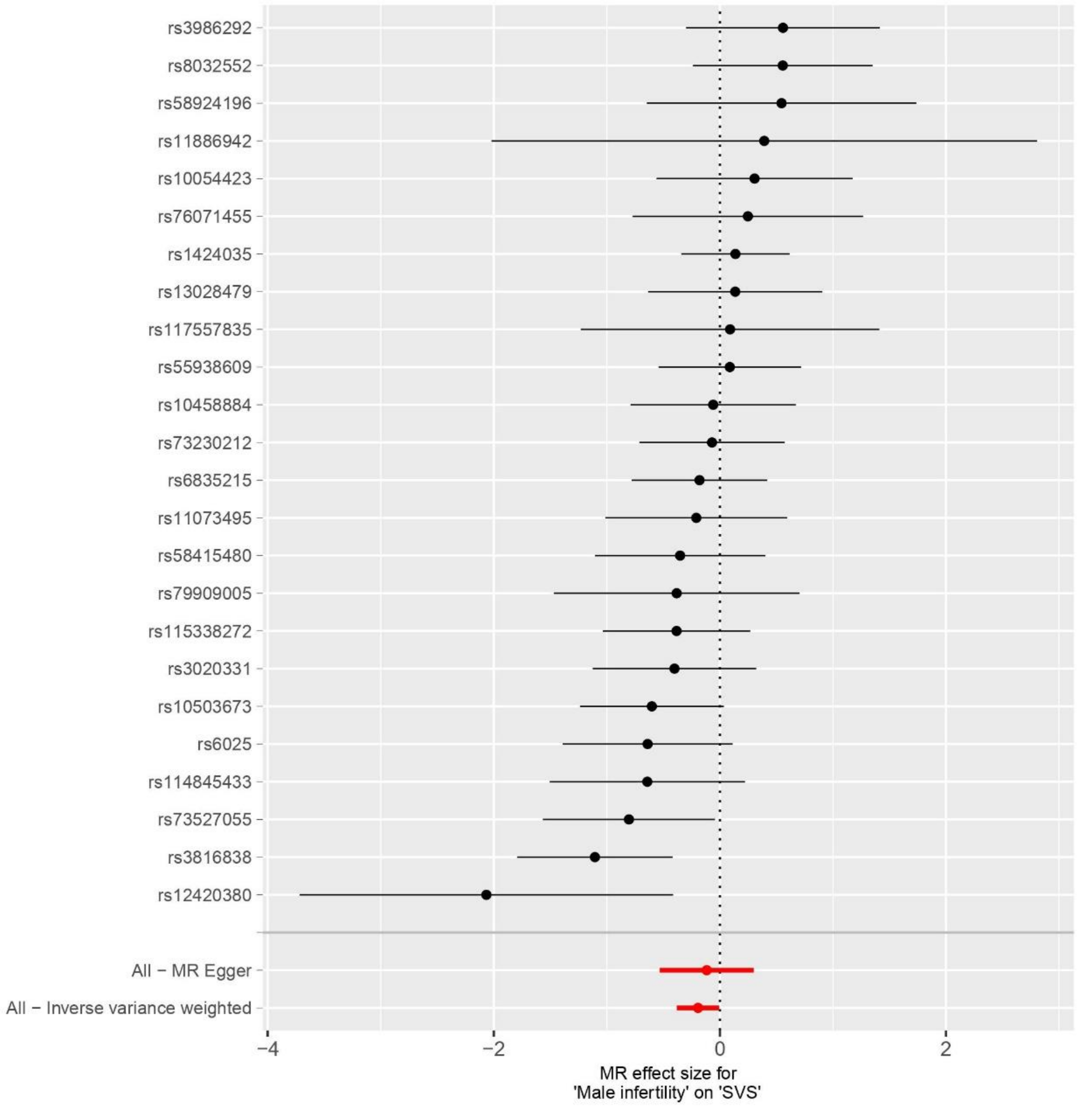

Figure S5. Forest plot of effect estimates for the causal relationship between male infertility and small vessel stroke risk at each SNP level. SVS, small vessel stroke; MR, Mendelian randomization; SNP, single nucleotide polymorphism.
